# Innate-like T cell subset commitment in the murine thymus is independent of TCR characteristics and occurs during proliferation

**DOI:** 10.1073/pnas.2311348121

**Published:** 2024-03-26

**Authors:** Vadim K. Karnaukhov, Anne-Laure Le Gac, Linda Bilonda Mutala, Aurélie Darbois, Laetitia Perrin, Francois Legoux, Aleksandra M. Walczak, Thierry Mora, Olivier Lantz

**Affiliations:** ^a^Institut Curie, Paris Sciences & Lettres University, Inserm U932, Immunity and Cancer, Paris 75005, France; ^b^Laboratoire de Physique de l’École Normale Supérieure, Paris Sciences & Lettres University, CNRS, Sorbonne Université and Université Paris Cité, Paris 75005, France; ^c^INSERM Equipe de Recherche Labellisée 1305, CNRS UMR6290, Université de Rennes, Institut de Génétique & Développement de Rennes 35000, France; ^d^Laboratoire d’Immunologie Clinique, Département de médecine diagnostique et théranostique, Institut Curie, Paris 75005, France; ^e^Centre d’Investigation Clinique en Biothérapie Gustave-Roussy Institut Curie (CIC-BT1428), Paris 75005, France

**Keywords:** MAIT, TCR, development, thymus, subset

## Abstract

How T-cell receptor (TCR) characteristics determine subset commitment during T-cell development is still unclear. Here, we addressed this question for innate-like T cells, mucosal-associated invariant T (MAIT) cells, and invariant natural killer T (iNKT) cells. MAIT and iNKT cells have similar developmental paths, leading in mice to two effector subsets, cytotoxic (MAIT1/iNKT1) and IL17-secreting (MAIT17/iNKT17). For iNKT1 vs iNKT17 fate choice, an instructive role for TCR affinity was proposed but recent data argue against this model. Herein, we examined TCR role in MAIT and iNKT subset commitment through scRNAseq and TCR repertoire analysis. In our dataset of thymic MAIT cells, we found pairs of T-cell clones with identical amino acid TCR sequences originating from distinct precursors, one of which committed to MAIT1 and the other to MAIT17 fates. Quantitative in silico simulations indicated that the number of such cases is best explained by lineage choice being independent of TCR characteristics. Comparison of TCR features of MAIT1 and MAIT17 clonotypes demonstrated that the subsets cannot be distinguished based on TCR sequence. To pinpoint the developmental stage associated with MAIT sublineage choice, we demonstrated that proliferation takes place both before and after MAIT fate commitment. Altogether, we propose a model of MAIT cell development in which noncommitted, intermediate-stage MAIT cells undergo a first round of proliferation, followed by TCR characteristics-independent commitment to MAIT1 or MAIT17 lineage, followed by an additional round of proliferation. Reanalyzing a published iNKT TCR dataset, we showed that this model is also relevant for iNKT cell development.

## Significance

T-cell receptor (TCR) binding to its ligand(s) is essential for T-cell development into several distinct lineages. The strength of this interaction and physicochemical characteristics of TCR were previously shown to govern commitment to particular fates (e.g., T cells with hydrophobic TCRs preferentially acquire regulatory phenotype). It is not clear whether this conclusion applies to other T cell subsets. Here, we investigated the role of TCR characteristics in the fate choice of evolutionarily conserved innate-like T cells. We propose a model of their development including two rounds of proliferation before and after lineage commitment, which is independent of TCR characteristics. Our framework may also help to determine the role of TCR characteristics in subset choice for other T-cell lineages.

During their development in the thymus, T cells differentiate into several lineages (e.g., CD4+, CD8+, and regulatory T cells) according to the signals they receive through TCR during positive selection. TCR characteristics influence commitment to particular T cell subsets. For example, hydrophobic TCRs promote commitment to regulatory T cell (Treg) rather than to conventional T (Tconv) fate (1–3). Comparison of TCR repertoires of CD4+ and CD8+ T cells evidenced prominent differences, which can be used to predict CD4+ or CD8+ phenotype of a T cell based on its TCR sequence (4–7). However, it is not clear whether TCR characteristics also govern fate choice for other T cell lineages. In this study, we investigated the role of TCR characteristics in fate choice of innate-like T cells—mucosal-associated invariant T (MAIT) cells and invariant natural killer T (iNKT) cells—which differentiate in the thymus into two distinct sublineages, type 1 (MAIT1/iNKT1) and type 17 (MAIT17/iNKT17).

MAIT cells are evolutionarily conserved innate-like T cells that recognize metabolites (5-OE-RU and 5-OP-RU) of the microbial vitamin B2 biosynthetic pathway presented by the nonpolymorphic MHC class Ib molecule, MR1. 5-OE-RU and 5-OP-RU are produced by most bacterial species but not by mammalian cells, suggesting an important role for MAIT cells in antibacterial immunity (8, 9). Moreover, MAIT cells are involved in tissue repair (e.g., after bacterial infection or wound) (10, 11) and antiviral responses (12) [although viruses do not produce MAIT ligands, MAIT cells can be activated in a TCR-independent manner to exert effector functions (13)].

MAIT cells are very abundant in humans: up to 10% of T cells in the peripheral blood and up to 40% in some other organs (skin or liver) (14, 15). In mice, MAIT cells are less abundant: <1% of T cells in the blood and up to 5% in some tissues (lung, gut lamina propria, or skin) (16–18). In both species, the reactivity towards MR1 loaded with 5-OP/E-RU is associated with the use of a semi-invariant T-cell receptor (TCR) composed of an almost invariant TCRα chain (containing TRAV1 and TRAJ33 genes in mice and a CDR3α length of 14 amino acids) associated with more variable TCRβ chains preferentially using particular TRBV genes (e.g., TRBV13 and TRBV19 in mice) (19). Importantly, both TRAV1 and MR1 are exceptionally conserved in mammals with a coevolution of the two loci across species and a strong signal of positive selection indicating important and nonredundant functions of the MR1-restricted TRAV1^+^ MAIT cells (20).

Similarly to MAIT cells, iNKT cells recognize nonpeptidic antigen [glycolipid alpha-Galactosylceramide (α-GC)] presented by an MHC-like molecule (CD1d), using TCR with invariant α- (with TRAV11 and TRAJ18 in mice) and restricted β-chains (using mostly TRBV1, TRBV29 and TRBV13 genes in mice). iNKT cells are highly abundant in mice (~10% in the liver). MAIT and iNKT cells share a common developmental path with conventional T cells (Tconv) up to the CD4^+^CD8^+^ double positive (DP) thymocyte stage (21). However, in contrast to Tconv which are positively selected by classical MHC molecules expressed by thymic epithelial cells, MAIT and iNKT cells are positively selected by DP thymocytes expressing MR1 and CD1d. TCR triggering together with homotypic SLAM interactions activate a SAP-dependent signaling pathway leading to the acquisition of a particular transcriptional program allowing direct tissue migration (22, 23). Importantly, all stages of innate-like T cell development take place in the thymus where MAIT and iNKT cells acquire their effector functions and the ability to migrate to nonlymphoid tissues (17).

In mice, MAIT and iNKT cells encompass two effector subsets, MAIT1/iNKT1 and MAIT17/iNKT17 cells, that are somewhat similar to Th1 and Th17 CD4^+^ Tconv cells, respectively (17, 24). MAIT1 and iNKT1 cells express the Tbet transcription factor, secrete IFNγ, and are cytotoxic, while MAIT17 and iNKT17 cells mainly express tissue repair mediators (11, 13). MAIT1/iNKT1 and MAIT17/iNKT17 subsets also differ regarding their tissue location: MAIT1 and iNKT1 cells are preferentially found in the liver and spleen while MAIT17 and iNKT17 cells are in the lungs and skin (17). An iNKT2 subset has also been described but whether it represents a development intermediate or a defined subset is still debated (25), while the existence of a MAIT2 subset is unclear (17, 26).

The intrathymic processes governing MAIT cell fate choice to either MAIT1 or MAIT17 subsets remain largely unknown but insights into this process can be obtained through comparison with the development of iNKT cells. Following analysis of TRBV gene usage in the iNKT1/2/17 subsets (27), it was suggested that iNKT TCR specificity may be involved in iNKT subset choice (28). Moreover, CD1d:α-GC tetramer staining intensity, which correlates with TCR avidity, was measured in developing iNKT cells and was associated with iNKT subsets suggesting a role for TCR avidity in subset choice (28). Agonist signaling seemed to be of decreasing intensity from iNKT2 to iNKT17 and iNKT1 subsets. Accordingly, decreasing TCR agonist signaling through the use of a hypomorphic allele of Zap70 led to a decrease in iNKT2 and iNKT17 subset proportion (28, 29). Finally, when iNKT TCRs with different avidities to CD1d:α-GC were reexpressed in retrogenic mice, the proportion of iNKT subsets within the clones was somewhat correlated with the avidity of the TCRs, suggesting an instructive affinity-based model of iNKT subset choice (30).

In contrast, a stochastic model was recently proposed based on the results of an experiment in which transient induction of the iNKT TCRα chain rearrangement in DP thymocytes led to a synchronous wave of iNKT cell development including both iNKT1 and iNKT17 cells (31). As the homogeneous TCR signal lasted less than 24 h and the iNKT1/17 lineage commitment took place 5 to 6 d later, the iNKT1/17 choice was unlikely to be instructed by TCR characteristics. However, as the TCRβ was not controlled in this experimental model, a role for TCR affinity in iNKT lineage choice cannot be formally ruled out. Thus, whether iNKT1/iNKT17 subset choice is instructed by TCR characteristics or stochastic is still a matter of debate. Regarding MAIT cells, whether TCR affinity to its exogenous or endogenous MR1-restricted antigen impacts subset choice and at which stage of MAIT cell development this choice happens are both unclear.

In this study, we addressed this question by comparing the TCR repertoires of MAIT1 and MAIT17 cells from the mouse thymus using 5′ scRNAseq and scTCRseq of MR1:5-OP-RU tetramer–positive thymocytes. A thorough analysis of TCR features between MAIT subpopulations did not show any difference between the repertoires of MAIT1 and MAIT17 subsets. Quantitative simulation of clonotype distributions of MAIT1 and MAIT17 cells allowed us to investigate the role of TCR characteristics in MAIT fate choice, and to pinpoint the stage at which lineage commitment occurs. Our results indicate that the TCR characteristics are not instructive in MAIT lineage choice and that MAIT1/17 commitment takes place during MAIT cell proliferation in the thymus. Finally, we performed analogous analysis of a published scRNA-seq and scTCR-seq dataset of iNKT cells from mouse thymus and demonstrated that our conclusions are also relevant for iNKT1 vs. iNKT17 fate commitment.

## Results

### Single-Cell RNA-Seq and TCR-Seq Dataset of MAIT Cells from Mouse Thymus.

**Fig. 1.**
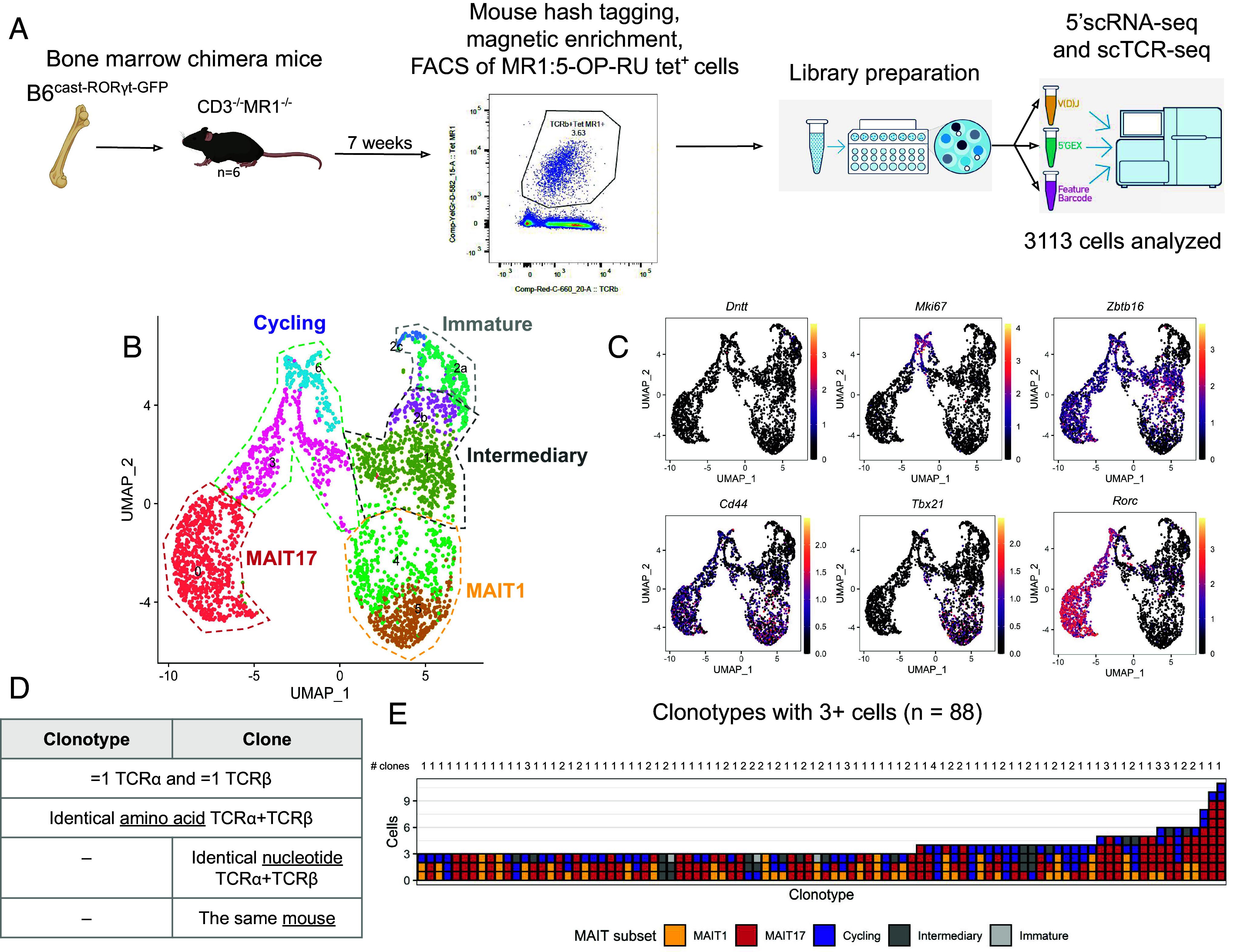
Overview of the experimental dataset. (*A*) Scheme of the experiment: single-cell RNA and TCR sequencing of MR1-5:OP-RU tetramer-positive cells from the thymus of bone marrow chimera mice. (*B*) UMAP of the scRNA-seq dataset of MR1-5:OP-RU tetramer-positive cells. (*C*) Expression levels of marker genes of MAIT subpopulations, projected to the UMAP plot. (*D*) Definition of clonotypes and clones. (*E*) MAIT subset composition of the most expanded (≥3 cells) clonotypes in the dataset. The number of clones comprising the clonotypes is shown on the top.

MAIT cells derive from DP thymocytes which have rearranged a TCR recognizing 5-OP-RU presented by MR1. Such DP thymocytes can give rise to two types of cells: either bona fide MAIT cells (which further diverge to MAIT1 and MAIT17 subsets) or mainstream-like T cells. This choice depends on which type of MR1-expressing cells participated in the positive selection: DP thymocytes for MAIT cells and thymic epithelial cells (TECs) for mainstream-like T cells (21, 23). To focus on bona fide MAIT cells and to deplete confounding mainstream-like MR1:5-OP-RU tetramer+ T cells, we generated bone marrow chimeras in which bone marrow from MR1+ B6 mice was injected into sublethally irradiated *Mr1*^−/−^*CD3e*^−/−^ mice. In these mice, MR1 is expressed exclusively by hematopoietic cells and consequently, only bona fide MAIT cells can develop from MR1:5-OP-RU-recognizing T cell precursors. After 7 wk of reconstitution, we isolated MR1:5-OP-RU tetramer+ T cells from the thymus of 6 bone-marrow chimeras using magnetic bead enrichment and FAC sorting (*Methods*). The cells from each individual chimeric mouse were hash-tagged to reflect the mice of origin and then were subjected to 10× 5′ transcriptomic and VDJ sequencing (Fig. 1*A*). After quality control, 3,113 cells with a median gene expression of 2,714 were retained for downstream analysis. We then performed uniform manifold approximation and projection (UMAP) (32) and unsupervised graph-based clustering of transcriptome data to partition MAIT cells into five subsets (Fig. 1*B*). These subsets were named according to the expression of key genes (Fig. 1*C*): immature (*Cd24a*, *Dntt*, *Rag1/2*), intermediate-stage (no *Cd24a* and no *Zbtb16* expression), cycling (*Mki67*), MAIT1 (*Tbx21*, *Nkg7*), and MAIT17 (*Rorc*, *Cxcr6*, *Il23r*).

For further analysis, we selected only cells for which exactly one TCRβ chain was detected paired with either one or two TCRα chains (in the case of two TCRα chains, only the one with TRAV1 was considered). In total, our dataset included 1,822 cells, of which 430 were mature MAIT1, 461 mature MAIT17 and 931 MAIT cells of earlier developmental stages (immature, intermediate and cycling) (*SI Appendix*, Fig. S1*A*). Twenty-eight cells expressing two TCRα chains were included in the analysis (*Methods*) and were found at similar frequency in MAIT1 and MAIT17 subsets (*SI Appendix*, Fig. S1*B*), indicating that dual TCRα expression does not bias MAIT fate choice.

### Assignment of Clonotypes and Clones.

For TCR analysis we assigned cells from our dataset into clonotypes and clones (Fig. 1*D*). We defined a clonotype as a set of cells having identical amino acid sequences of both TCRα and TCRβ chains. Since for TCR–ligand interaction only amino acid (but not nucleotide) sequence matters, all cells within a clonotype have the same TCR properties. A clone was defined as a set of cells having identical nucleotide sequences of both TCRα and TCRβ chains and originating from the same mouse. We assume that all cells within the clone originated from a single precursor MAIT cell since the probability of convergent recombination of the same TCR nucleotide sequence in the same mouse is very low (generation probabilities of individual MAIT TCR nucleotide sequence are in range 2 × 10^−43^ to 3 × 10^−12^ while the total number of T cells in mice is of order of 10^6^). Importantly, a single clonotype may be composed of several clones. Clonotypes and clones were annotated as “MAIT1” (included ≥1 MAIT1 cells and no MAIT17 cells), “MAIT17” (≥1 MAIT17 and no MAIT1), “MAIT1-MAIT17” (≥1 MAIT17 and ≥1 MAIT1) or “other” (only immature, intermediate-stage or cycling cells).

In total, we identified 1,392 different clonotypes, of which 274 encompassed two or more cells and 88 had at least three cells (Fig. 1*E*). The majority of identified clones encompassed exclusively MAIT1 or MAIT17 cells with an admixture of cycling and intermediate-stage cells (Fig. 1*E*). The highest level of expansion (mean clone size in a given subset) was observed in the MAIT17 subset, in line with previous observations of active proliferation of MAIT17 cells (23, 33) (*SI Appendix*, Fig. S1*C*). For MAIT1 cells, intermediate and cycling subsets the level of expansion was approximately at the same level, while for immature MAIT cells, almost no expansions were observed (*SI Appendix*, Fig. S1*C*) as expected (23).

Although the majority of clonotypes were either MAIT1 or MAIT17, we also detected several MAIT1-MAIT17 clonotypes (Fig. 1*E*). One might think that this fact alone can indicate that TCR is not instructive in fate commitment. However, hypothetical scenarios with TCR-governed fate choice (e.g., with high and low affinity signals inducing MAIT1 and MAIT17 lineage commitment, respectively) may also result in the presence of TCRs with the same sequence in both MAIT1 and MAIT17 subsets (*SI Appendix*, Fig. S2). For a scenario in which a single MR1 ligand selects MAIT cells, precursors with intermediate TCR affinity may probabilistically commit to MAIT1 or MAIT17 fate (*SI Appendix*, Fig. S2*A*). In the case of two different MR1-bound ligands, a single TCR may have different affinities for them (high for one and low for another) leading to either a MAIT1 or MAIT17 fate depending on the selecting ligand (*SI Appendix*, Fig. S2*B*).

### TCR Characteristics Are Not Instructive to MAIT Sublineage Choice.

**Fig. 2.**
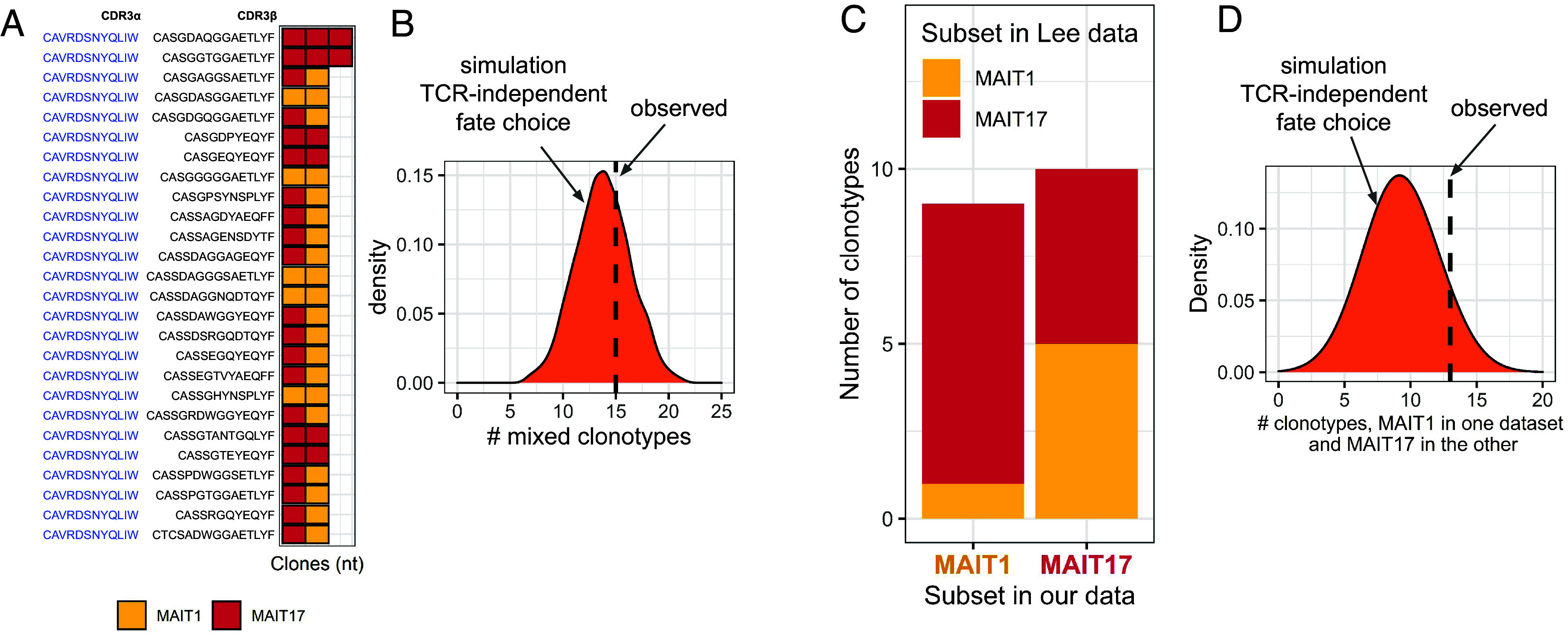
MAIT1 vs. MAIT17 fate choice is independent of TCR characteristics. (*A*) Clonotypes encompassing several clones. Only MAIT1 and MAIT17 clones were considered. Clonotypes with a unique amino acid sequence are shown on the *Y* axis. All clonotypes have canonical MAIT CDR3α (blue). The *X* axis shows distinct clones within the clonotype having identical amino acid sequences but distinct nucleotide sequences or mouse of origin. Each clone is shown in a square, the color corresponds to MAIT subsets. (*B*) Label shuffling simulation showing the expected number of clonotypes made of both MAIT1 and MAIT17 clones on the assumption of TCR-characteristics-independent MAIT lineage choice. (*C*) Clonotypes which are found both in our and Lee datasets. Each bar represents one subpopulation in our dataset. The colors represent the subset identity of these clonotypes in the Lee dataset. (*D*) Label shuffling simulation showing the expected number of clonotypes assigned to different subsets in our and Lee datasets in the assumption of TCR-independent MAIT lineage choice. In panels *B* and *D* the number observed in our dataset is shown in a dashed line.

To test whether TCR characteristics are instructive for MAIT fate choice, we focused on clonotypes which are composed of several clones. These distinct clones within a single clonotype may be regarded as “biological replicates”: They have the same TCR protein sequence with identical ligand specificity and affinity, but have been derived from different precursor cells and selected independently. In our dataset, we found 26 clonotypes which encompassed 2 (n = 24) or 3 (n = 2) distinct clones (MAIT1 or MAIT17). In Fig. 2*A*, each such clonotype with a unique amino acid sequence is shown in a separate row; each square represents a single clone with color indicating its subset identity (MAIT1 or MAIT17). We observed that 15 out of these 26 clonotypes are “mixed,” encompassing simultaneously both MAIT1 or MAIT17 clones. To assess the meaning of this number regarding a putative role of TCR in MAIT fate choice, we performed in silico simulations with random shuffling of subset identity labels for clones (keeping the subset ratio and size distribution fixed). This simulation reflects the situation in which the fate of each clone is chosen stochastically, independently of its TCR amino-acid sequence. We repeated this random shuffling 1,000 times. After every simulation we calculated the number of mixed clonotypes, which ranged from 9 to 19 (95% CI, median 14; Fig. 2*B*). The number observed in our data (n = 15) fits well within this interval, indicating that TCR independent lineage commitment is compatible with our data.

Another way to track biological replicates is to search for the same amino acid TCR sequence in different datasets. Recently another dataset of scRNAseq and scTCRseq of MAIT cells from mouse thymus was published by Lee et al. (further referred to as “Lee dataset”) (26). To check whether these data obtained from BALB/c mice are comparable with our data from B6 mice, we performed integration of the two datasets (*SI Appendix*, Fig. S3*A*). This analysis shows that our and Lee datasets fully overlap (*SI Appendix*, Fig. S3*B*), indicating shared gene expression patterns and identical properties of MAIT subsets (*SI Appendix*, Fig. S3 *C* and *D*) in B6 and BALB/c mice. We searched the Lee dataset for clonotypes found in our dataset and identified 19 clonotypes shared between both. If the TCR amino-acid sequence governed cell fate, we would expect clonotypes with identical sequences to have the same MAIT subset identity in both datasets. However, this was not the case: Among the 9 shared sequences that were in the MAIT1 subset of our dataset, only 1 was also found in the MAIT1 subset of the Lee dataset, while 8 sequences belonged to the Lee MAIT17 subset (Fig. 2*C*). For the 10 shared sequences that were in the MAIT17 subset of our dataset, only 5 were also in the MAIT17 subset of the Lee dataset, and the other 5 were in the Lee MAIT1 subset (Fig. 2*C*). Overall, only 6 clonotypes were found in the same subset in both datasets while 13 were in the MAIT1 subset of one dataset while being in the MAIT17 subset of the other. We again performed label shuffling simulations, reflecting stochastic MAIT fate choice. The simulation showed that the number of clonotypes assigned to the different subsets in our and Lee dataset is expected to range from 3 to 16, which fits with the value (n = 13) we observed (Fig. 2*D*).

Altogether, these results indicate that MAIT subset choice is stochastic and independent of TCR characteristics.

### MAIT1 and MAIT17 Are Clustered Together Based on TCR Sequence Similarity.

**Fig. 3.**
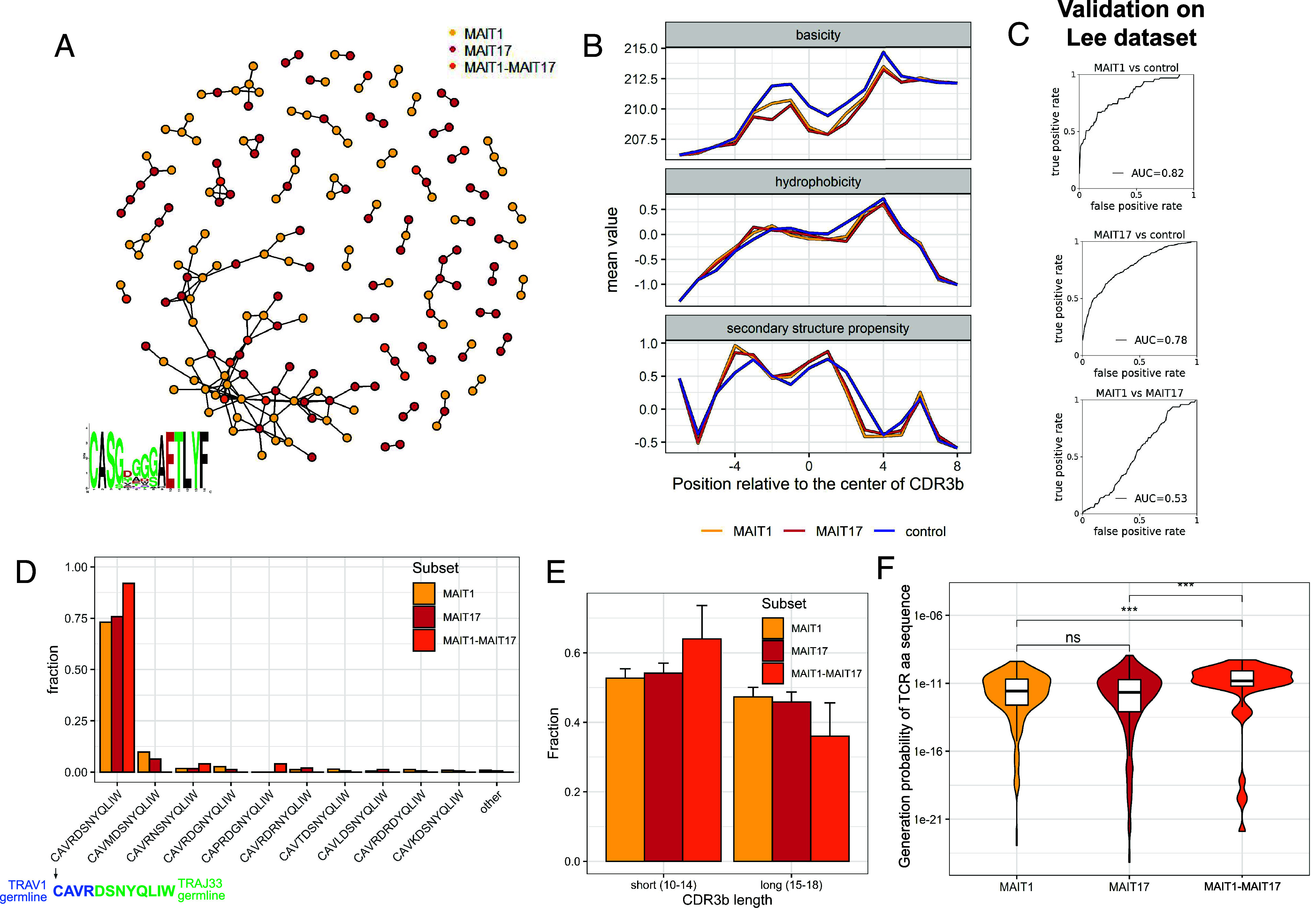
MAIT1 and MAIT17 clonotypes have similar TCR characteristics. (*A*) CDR3β sequence similarity graph for MAIT1 and MAIT17 clonotypes. Each dot (vertex) represents a single clonotype, with color indicating the subset. Vertices are connected with a line (edge) if they differ by a single amino acid. Note that MAIT1 and MAIT17 TCRs are mixed together in the largest clusters. *Inset*: logo plot for CDR3β of the largest cluster (n = 60 sequences). Only sequences with canonical TRAV1 and CDR3α CAVRDSNYQLIW are considered. (*B*) Physicochemical profiles of CDR3β sequences for MAIT1 and MAIT17 clonotypes in comparison with control clonotypes from the blood of healthy mice from ref. 35. Position in CDR3β is shown relative to the center of CDR3β to enable consideration of CDR3β of different lengths. For each position the average value of the considered physicochemical property (basicity, hydrophobicity, or secondary structure propensity) was calculated for all sequences in the group and plotted on the *Y* axis. (*C*) Receiver operating characteristic (ROC) curves of a classifier based on SONIA software (36) to distinguish MAIT1 vs. MAIT17, MAIT1 vs. control and MAIT17 vs. control clonotypes. AUC = area under ROC curve. (*D*) Fractions of different CDR3α sequences in MAIT subsets. Note that the most frequent CDR3α sequence does not require nontemplate nucleotide additions during VJ-recombination (germline-encoded CDR3α fragments coming from TRAV1 and TRAJ33 genes are highlighted in blue and green, respectively). (*E*) CDR3β length distribution in MAIT subsets. (*F*) Distribution of generation probabilities of TCRα+TCRβ amino acid sequences in different subsets. Note that TCR sequences shared between MAIT1 and MAIT17 have higher generation probability. ***P-value < 0.001, ns: not significant. Only sequences with exactly 1 TCRα and 1 TCRβ are considered.

We then explored the TCR sequence similarity of MAIT1 and MAIT17 clonotypes. First, we compared Vα (*SI Appendix*, Fig. S4*A*) and Vβ gene usage (*SI Appendix*, Fig. S4*B*), which was similar for MAIT1 and MAIT17 clonotypes. Then, we analyzed the similarity of MAIT1 and MAIT17 CDR3β sequences. For this, we identified components (clusters) in a graph where each vertex (dot) corresponds to a single clonotype and vertices are connected with an edge (line) if they differ by only a single amino acid (Fig. 3*A*). If MAIT1 and MAIT17 cells have different specificities or different affinities to the same ligand, their sequences should cluster separately (34). The largest observed cluster contained 60 sequences (34 MAIT1, 22 MAIT17, and 4 MAIT1-MAIT17) with a consensus CDR3β motif CASGDGGGAETLYF and several clusters contained 2 to 7 sequences (Fig. 3*A*). Notably, all the clusters with a size >4 sequences encompassed both MAIT1 and MAIT17 clonotypes (Fig. 3*A*).

Finally, we performed hierarchical clustering using the tcrdist3 package (37). tcrdist3 calculates pairwise distances between full TCR sequences (both TCRα and TCRβ sequences are considered) as the sum of weighted Levenstein distances between their CDR amino acid sequences, assigning three-times higher weights to CDR3 mismatches compared to CDR1 and CDR2 and taking into account the evolutionary conservation of the substitutions (e.g., weights of L→I and L→K mismatches are 2 and 4, respectively). The resulting dendrogram of MAIT sequences indicated that no clusters specific to MAIT1 or MAIT17 clonotypes could be identified (*SI Appendix*, Fig. S5).

Taken together, these results demonstrate that MAIT1 and MAIT17 cells have similar TCR sequences clustering together, suggesting that both subsets have the same antigen specificity.

### MAIT1 and MAIT17 Clonotypes Cannot be Distinguished Based on CDR3 Sequence.

We then compared the general characteristics of CDR3β sequences of MAIT1 and MAIT17 clonotypes and asked whether they can be distinguished. As a control, we used a dataset of CDR3β sequences found in conventional T cells from mouse peripheral blood described by Sethna et al. (35). First, we compared the profiles of amino acid physicochemical properties (basicity, hydrophobicity, and propensity to form secondary structure) along CDR3β sequences (Fig. 3*B*). These profiles were similar for MAIT1 and MAIT17 clonotypes but differed substantially between MAIT and conventional T cells: Both MAIT1 and MAIT17 CDR3β have lower basicity for the central positions as compared to control, lower hydrophobicity for N-end part, higher hydrophobicity for C-end part and varying propensity to form α-helices across the sequence (Fig. 3*B*).

To investigate whether MAIT1 and MAIT17 clonotypes can be distinguished based on their CDR3β sequences, we constructed a classifier based on the SONIA software (6, 36) that demonstrated high performance in distinguishing TCRs with different specificities in a recent benchmark study (38). This classifier uses a one-hot encoding of CDR3β amino acid sequence and then fits a linear regression model using a regularization for the feature selection. The validation of the classifier’s accuracy was performed on an independent test dataset from Lee et al. (26).

To confirm that the SONIA classifier is suitable for this task, we first trained it to distinguish between MAIT1 and control CDR3β or between MAIT17 and control CDR3β. In both settings, the classifier had a high performance with area under receiver operator curve (ROC AUC) around 0.8 (Fig. 3*C*). Then we trained the same classifier to distinguish MAIT1 from MAIT17 clonotypes. Here, the performance was close to random with ROC AUC equal to 0.53 (the value 0.5 corresponds to random predictions; Fig. 3*C*). We also performed validation of the classifier using fivefold cross-validation (CV) on our dataset which demonstrated similar performance (*SI Appendix*, Fig. S6*A*). To demonstrate the robustness of our results, we repeated the analysis with an alternative control dataset of TCR sequences from healthy mice from the Chudakov lab [https://zenodo.org/record/​6339774#.ZAikGXbMJPa]. We obtained the same patterns in CDR3β physicochemical profiles (*SI Appendix*, Fig. S6*B*). The ROC AUC of the SONIA classifier in distinguishing MAIT1/MAIT17 and Chudakov control sequences was also around 0.8 (*SI Appendix*, Fig. S6*C*) while sequences from Chudakov and Sethna datasets could not be distinguished between each other (ROC AUC 0.51; *SI Appendix*, Fig. S6*D*).

Finally, we looked for differences in sequence motifs between MAIT1 and MAIT17 CDR3β sequences (*SI Appendix*, Fig. S7*A*). To remove V(D)J recombination bias, we calculated the enrichment of positional amino acid usage relative to a synthetic TCR repertoire generated by OLGA software (39). As a metric of enrichment/depletion, we used the weights of the SONIA classifier. The sequence patterns of MAIT1 and MAIT17 CDR3β were similar, with enrichment of glycines in several positions in the center and AELTY motif in the C-terminal part (*SI Appendix*, Fig. S7*B*).

In summary, MAIT1 and MAIT17 clonotypes have similar profiles of amino acid sequence in their CDR3β (both in terms of physicochemical properties and amino acid identity) and cannot be distinguished based on them. Meanwhile, MAIT clonotypes have specific sequence features which differentiate them from other TCRs from conventional T cells.

### MAIT1-MAIT17 Clonotypes Have Higher Generation Probability.

We then explored whether TCR sequences of MAIT1-MAIT17 clonotypes have some features distinguishing them from clonotypes exclusively made of MAIT1 or MAIT17 cells. In terms of V gene usage, MAIT1, MAIT17, and MAIT1-MAIT17 clonotypes displayed similar profiles: Almost all of them have TRAV1 characteristic of MAIT cells (*SI Appendix*, Fig. S4*A*) with preferential usage of certain TRBV segments (TRBV13-3, TRBV13-2, TRBV19) (*SI Appendix*, Fig. S4*B*), in concordance with previous reports (19). Regarding CDR3α, one particular sequence was the most abundant, CAVRDSNYCLIW, found in 74% of MAIT1 and 75% of MAIT17 clonotypes (Fig. 3*D*). For MAIT1-MAIT17 clonotypes, the prevalence of that CDR3α sequence was even higher, 89% (Fig. 3*D*). This sequence is relatively easily generated in the process of VJ recombination as it is constructed of non-modified germline-encoded parts of TRAV1 and TRAJ33 genes (Fig. 3*D*) without requirement of nontemplate nucleotide additions.

As CDR3β sequences were much more variable than CDR3α, we analyzed CDR3β length distributions in the different subsets (*SI Appendix*, Fig. S8). For MAIT1-MAIT17 CDR3β, this distribution was shifted to shorter lengths: The fraction of short sequences (10 to 14 amino acids) was 70% for MAIT1-MAIT17 CDR3β, while for MAIT1 and MAIT17 CDR3β this value was 53% and 54%, respectively (Fig. 3*E*). Shorter CDR3 sequences are easier to generate by the VDJ recombination process because they require less nucleotide additions (36). Linking this observation with the above-mentioned higher prevalence of germline-encoded CDR3α in MAIT1-MAIT17 clonotypes, we can conclude that both TCRα and TCRβ sequences of MAIT1-MAIT17 clonotypes are skewed to easily generated sequences. To put this into numbers, we computed the generation probability (Pgen) of TCR sequences of MAIT clonotypes using the OLGA software (39). Pgen estimates the probability to generate TCR having exactly the same amino acid sequence using a model of V(D)J-recombination inferred from large datasets of nonproductive TCR sequences (40). Pgen did not differ between MAIT1 and MAIT17 clonotypes, but was significantly higher for MAIT1-MAIT17 clonotypes as compared to both MAIT1 and MAIT17 clonotypes (*P* < 0.001) (Fig. 3*F*). It should be noted that higher Pgen values correspond to higher probabilities to rearrange exactly the same amino acid sequence (with possibly different nucleotide sequence) in different precursor cells. This is illustrated by higher Pgen values for clonotypes that include several clones compared to the clonotypes composed of a single clone (*SI Appendix*, Fig. S9*A*). Moreover, no differences between MAIT1, MAIT17, and MAIT1-MAIT17 TCR Pgen values were found when comparing clonotypes composed of the same number of clones (*SI Appendix*, Fig. S9*A*).

Thus, the higher Pgen of MAIT1-MAIT17 clonotypes might be interpreted in the following way. Some MAIT TCR sequences (mostly the ones with high Pgen) are rearranged independently in several precursor cells. These cells then commit to either MAIT1 or MAIT17 subsets. Since fate choice is stochastic (as discussed above), for ~50% of cases two independent cells with the same TCR sequence commit to different fates, resulting in the appearance of a MAIT1-MAIT17 clonotype. Consequently, MAIT1-MAIT17 clonotypes are preferentially composed of TCR amino acid sequences with relatively high Pgen, rearranged independently in several precursor cells. To illustrate this, we calculated which fractions of MAIT1, MAIT17 and MAIT1-MAIT17 clonotypes are composed of 1, 2, or 3 clones and found that 50% of MAIT1-MAIT17 clonotypes consist of 2+ clones while for MAIT1 and MAIT17 this number does not exceed 5% (*SI Appendix*, Fig. S9*B*).

### MAIT Cells Proliferate Both Before and After Sublineage Commitment.

**Fig. 4.**
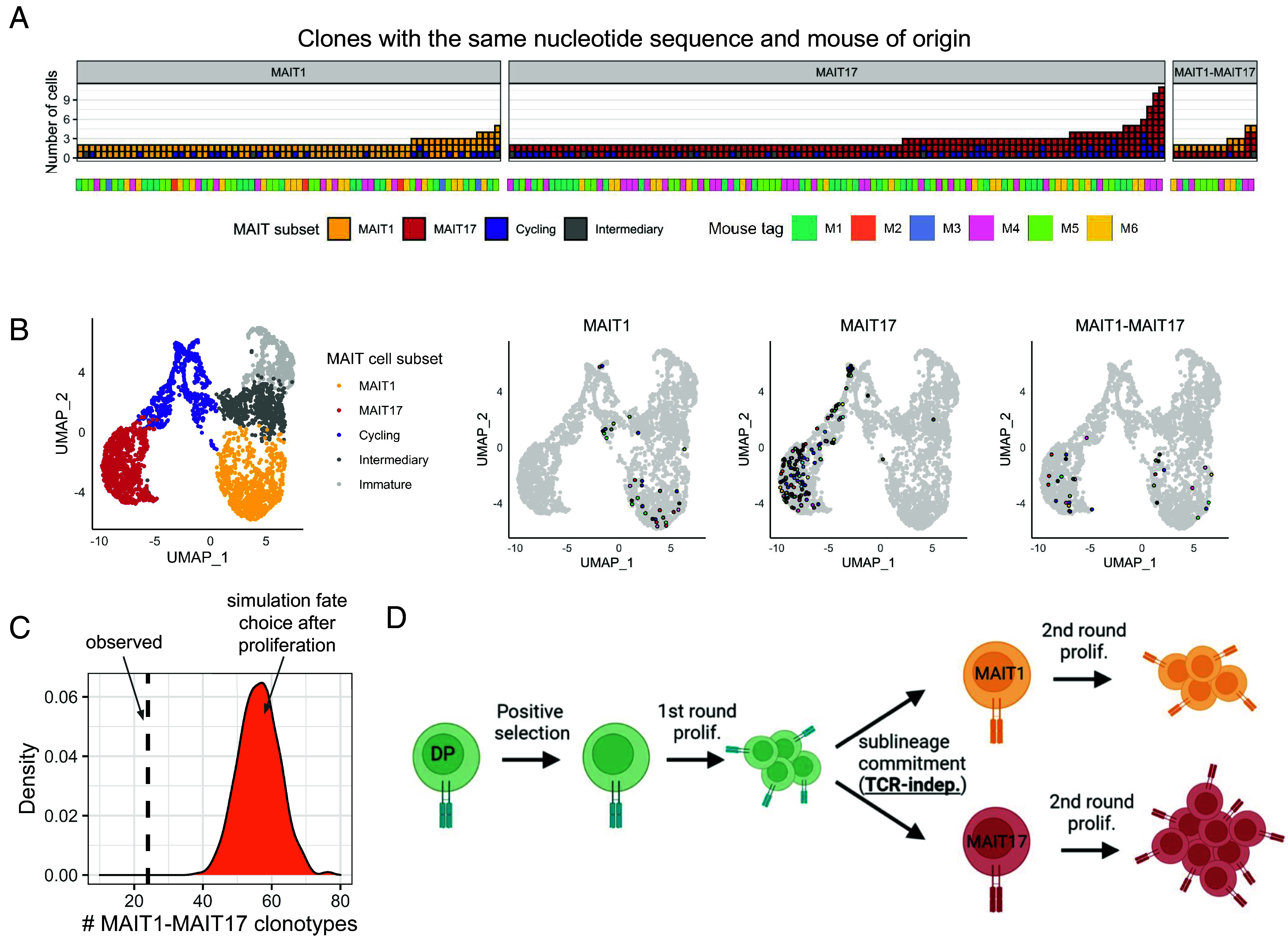
MAIT1 vs. MAIT17 lineage choice takes place during proliferation. (*A*) MAIT clones containing at least two cells with identical nucleotide sequences of TCR and originating from the same mouse. Each clone is shown as a vertical bar that contains several squares representing single cells. The color of the squares indicates the subset identity of the MAIT cell. The mouse of origin of the clone is color-coded in the panel below the barplot. (*B*) *Left* panel: reference UMAP of MAIT cells assigned to different subsets. Other three panels: UMAP coordinates of cells comprising the clones from panel *A*. Cells corresponding to the same clone are indicated by the same color. MAIT1 and MAIT17 clonotypes with 3+ cells and MAIT1-MAIT17 clonotypes with 2+ cells are shown. (*C*) Label shuffling simulation showing the expected number of MAIT1-MAIT17 clonotypes assuming that MAIT cell fate choice takes place independently in each mature MAIT cell, after proliferation. (*D*) Proposed model of MAIT differentiation with TCR characteristics-independent fate choice and two rounds of proliferation.

We then attempted to define at which stage of MAIT cell development the sublineage commitment takes place: before, during, or after proliferation. First, we explored the possibility of MAIT fate choice at the earliest stage of development, before all rounds of proliferation (a precursor cell first commits to some fate and then proliferates). In this scenario, all descendants of a single precursor should have the same subset identity. Thus, we expect to observe only MAIT1 and MAIT17 clones (with an admixture of cycling and intermediate-stage cells), but not MAIT1-MAIT17 clones. However, in our dataset, we identified 14 clones encompassing both MAIT1 and MAIT17 cells (Fig. 4 *A* and *B*). Pgen for these clones does not statistically deviate from the distribution of Pgens for other clones with a median value of 10^−14^ (*SI Appendix*, Fig. S10). It is thus highly unlikely that MAIT1-MAIT17 clones result from convergent V(D)J-recombination leading to identical TCR sequences in independent precursor cells. Moreover, the number of such clones is relatively high (n = 14). This clearly demonstrates that one precursor cell can give rise to both MAIT1 and MAIT17 cells, indicating that MAIT cells proliferate before lineage commitment.

Then we performed a simulation of MAIT fate choice at the latest stage of MAIT cell development, after all rounds of proliferation. For this we shuffled subset identity labels for all cells in our dataset, keeping the relative subset abundance. This simulation was repeated 1,000 times; for each iteration, we calculated the number of MAIT1-MAIT17 clonotypes for the resulting shuffled dataset. The distribution of these values (Fig. 4*C*) had a median of 56 (95% CI: 45 to 68). In our dataset, we observed only 25 MAIT1-MAIT17 clonotypes (*SI Appendix*, Fig. S11) which is significantly lower than expected if the fate choice takes place after proliferation. This result indicates that MAIT1 and MAIT17 cells proliferate after sublineage commitment. Additional evidence for MAIT cell proliferation both before and after fate choice comes from the fact that cells from the most proliferating cluster (cluster #6 in Fig. 1*B*) include intermediate-stage as well as mature MAIT1 and MAIT17 cells (*SI Appendix*, Fig. S12). Notably, the number of proliferating MAIT1 cells was lower compared to MAIT17 cells. These data together with a relatively high expansion level in the MAIT17 subset (*SI Appendix*, Fig. S1*C*) and our previous results (23) imply that MAIT17 cells proliferate more compared to MAIT1 after fate commitment.

Taken together, these results demonstrate that a first round of proliferation occurs right after MAIT positive selection, followed by lineage choice, followed by a second round of proliferation (Fig. 4*D*).

### TCR Role and Timing in iNKT Fate Choice.

**Fig. 5.**
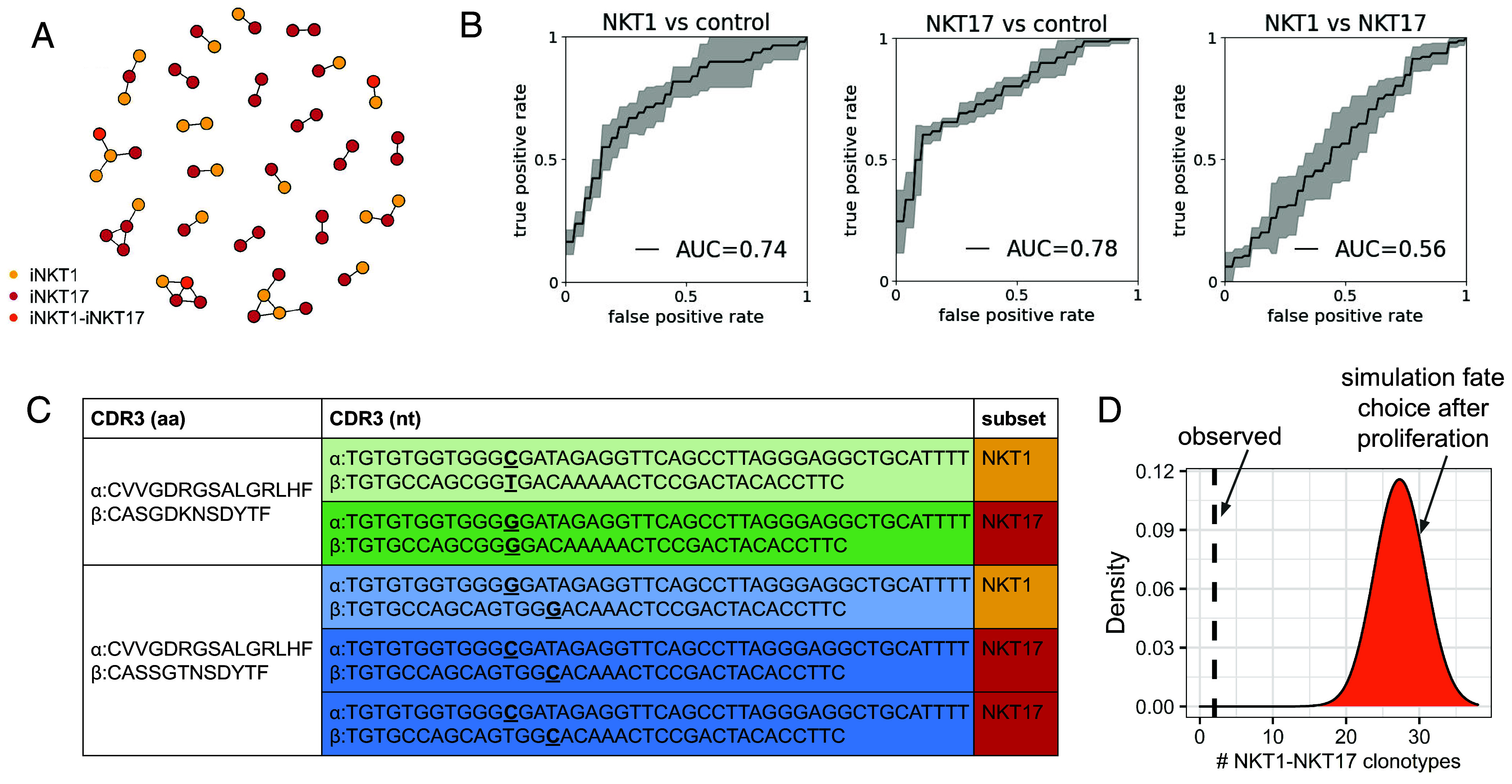
TCR role and timing in iNKT fate choice. (*A*) TCR sequence similarity of NKT1 (yellow), NKT17 (red), and NKT1-NKT17 (orange) clonotypes, analogous to Fig. 3*A*. (*B*) ROC curves for SONIA-based classifiers for distinction NKT1 vs. control, NKT17 vs. control, NKT1 vs. NKT17. (*C*) NKT1-NKT17 clonotypes encompassing two clones with distinct nucleotide sequences. Identical nucleotide sequences are shown in the same color. Clones within clonotypes differ in positions shown in bold and highlighted. (*D*) Simulation analogous to Fig. 4*C* indicating proliferation after iNKT fate commitment.

Finally, we investigated whether our model of MAIT cell development can also be applied to iNKT cells. We used scRNA-seq and scTCR-seq dataset of murine thymic iNKT cells from Lee et al. (26). In this dataset, more than 70% of clonotypes were assigned to the iNKT2 subset, which is now considered as an intermediate stage of iNKT cell development before commitment to either iNKT1 or iNKT17 fate (25). Only 182 and 137 clonotypes included at least one iNKT1 or one iNKT17 cell, respectively. First, we performed clustering analysis of iNKT1 and iNKT17 CDR3β sequences. We observed several large clusters of similar sequences (*SI Appendix*, Fig. S13*A*), where iNKT1 and iNKT17 clonotypes were mixed (Fig. 5*A*). In a dendrogram plotted based on pairwise tcrdist3 distances between CDR3β (37), the distribution of iNKT1 and iNKT17 clonotypes also did not show any pattern (*SI Appendix*, Fig. S13*B*). We next trained a SONIA-based (36) classifier and demonstrated that it could distinguish iNKT1 vs. control, iNKT17 vs. control, but not iNKT1 vs. iNKT17 (Fig. 5*B*). Moreover, despite the small number of iNKT1 and iNKT17 cells in the Lee dataset, we detected two cases of a pair of cells that had the same amino acid but different nucleotide sequences (clones within the clonotype). In both cases, clones committed to different fates: one to iNKT1 and the other to iNKT17 (Fig. 5*C*). In the Lee dataset from BALB/c mice, the majority of iNKT cells had been assigned to the iNKT2 subset which was previously considered as a terminally differentiated iNKT effector subset along with iNKT1 and iNKT17 subsets (27). However, recent studies based on scRNA-seq data analysis (25, 41) indicate that iNKT2 cells probably represent an intermediate stage of iNKT development being progenitors of iNKT1 and iNKT17 cells. We asked whether iNKT2 TCRs were distinguishable from either iNKT1 or iNKT17 TCRs according to their CDR3β sequence. For this, we trained the same SONIA-based classifier used above. The performance of the classifier was close to 0.5 both for iNKT2 vs. iNKT1 and iNKT2 vs. iNKT17 comparison (*SI Appendix*, Fig. S14*A*). We also trained the classifier to distinguish intermediate-stage MAIT cells [referred to as MAIT2 in Lee et al. (26)] from MAIT1 and MAIT17 cells, and for both comparisons the performance was also close to 0.5 (*SI Appendix*, Fig. S14*B*). Taken together, these results support TCR characteristics-independent lineage choice for iNKT analogously to MAIT cells. To pinpoint the timing of iNKT cell commitment, we performed the simulation of fate choice after proliferation. The number of iNKT1-iNKT17 clonotypes we observed in the experimental dataset (n = 2) is significantly lower than expected from the simulation (Fig. 5*D*), indicating a round of cell proliferation after iNKT fate choice. As we did not find any iNKT1-iNKT17 clones, we cannot strictly affirm proliferation before lineage commitment. However, this result is most likely explained by the scarcity of Lee dataset (only 42 clones with 2+ iNKT1 or iNKT17 cells). In summary, we conclude that our model of MAIT cell development (Fig. 4*D*) is also relevant for iNKT cells.

## Discussion

In this study, we investigated TCR role and timing in the innate-like T cell sublineage commitment using scRNA-seq and scTCR-seq data from mouse thymic MAIT and iNKT cells. We addressed two questions: 1) Are TCR characteristics instructive for MAIT1 vs. MAIT17 and iNKT1 vs. iNKT17 fate choice? 2) At what stage of MAIT and iNKT cell development (before, during, or after proliferation) does the lineage commitment take place?

For the first question, our analysis demonstrates that TCR characteristics are not instructive for MAIT and iNKT cell fate choice. Strong indication for this comes from the tracking of cells having identical TCR amino acid sequences but originating from different precursors (having different nucleotide sequences, or isolated from different mice, or identified in different studies). Such cells should have identical properties of their TCRs but their differentiation path was independent of each other. Our analysis shows that such cells stochastically commit to different subsets indicating that the characteristics of TCR are not instructive in lineage choice.

For the second question, we conclude that MAIT fate choice takes place during MAIT cell proliferation (in other words, proliferation takes place both before and after lineage commitment). The number of MAIT1-MAIT17 clonotypes observed in the experimental dataset was significantly lower than expected from in silico simulation of fate choice after proliferation, assuming that there is a round of proliferation after sublineage commitment. Proliferation before fate choice was indicated by existence of several (n = 14) events in which a single precursor gave rise both to MAIT1 and MAIT17 cells.

Conclusions of our work agree with the recent work of Bortoluzzi et al. (31) demonstrating that brief TCR signaling is not instructive for iNKT lineage choice. Still, previous reports suggested that TCR avidity may govern iNKT fate commitment (42). In particular, it was proposed that iNKT2 cells have higher avidity (which is a combination of affinity and TCR surface expression) towards their nominal ligand as compared to iNKT1 and iNKT17 cells (28). However, this fact could be explained by higher levels of TCR expression on iNKT2 cells owing to a specific gene expression program. Thus, higher avidity of iNKT2 cells may be a consequence but not a cause of iNKT fate choice. Increased Vβ7 usage in iNKT2 cells reported by Lee et al. (27) and Amable et al. (43) may be explained by preferential expansions of Vβ7 clones in the iNKT2 subset. It was previously shown that Vβ7 iNKT TCRs have higher affinity than Vβ8 TCRs (44). Since iNKT2 is the most proliferative iNKT subset (41), Vβ7-expressing iNKT cells are expected to form large clonal expansions in the iNKT2 subset (26), leading to increased Vβ7 usage. Notably, there is no difference reported between iNKT subsets in usage of Vβ8 which provides lower affinity for iNKT TCRs. This interpretation is also supported by the increased Vβ7 but not Vβ8 usage in CD1d+/- mice (43), which have a lower CD1d expression and thus favor selection of high-affinity iNKT cells. Depletion of iNKT2 in Zap70-hypomorphic mice (28, 29) could also be explained in line with our model. Deficiency in Zap70 leads to impairment of T cell proliferation and thus to decrease in the relative fraction of the most proliferative subset iNKT2 (41). Altogether, all previous results supporting TCR avidity-governed iNKT subset choice are also compatible with TCR-characteristics-independent fate commitment.

Our conclusions regarding two rounds of proliferation are in agreement with previous studies. For iNKT cells, Ki67 staining shows that a substantial fraction of cells is proliferating at all stages of development, from the most immature stage 0 to terminally differentiated stage 3 (45). In our dataset, we also detected cycling cells in committed MAIT1 and MAIT17 cells as well as in intermediary-stage MAIT cells. Integrated analysis of all available scRNA-seq datasets of MAIT and iNKT cells indicated a population of proliferating cells which are enriched for iNKT progenitor gene signature and express *Il13* (41). This population of cells was previously shown to be the common progenitor of iNKT1, iNKT2, and iNKT17 cells (46), further supporting iNKT cell proliferation before fate commitment.

The absence of biologically meaningful differences between TCR sequences of MAIT1 and MAIT17 cells is supported by the clustering of MAIT1 and MAIT17 TCR sequences together, the similarity of their CDR3β physicochemical profiles, and the failure of the SONIA classifier to distinguish them. It should be noted that different MAIT subsets might have subtle hallmarks that could not be robustly identified in a dataset with a relatively small size (i.e., correlated amino acid usage in different CDR3 positions). However, no such features that could not be detected by a SONIA classifier have been described in the literature so far for TCRs with other specificities. It is thus unlikely that MAIT1 and MAIT17 TCRs would display such hidden features.

The MAIT TCR sequence is characterized by the usage of TRAV1, TRAJ33, a conserved CDR3α of length 14, and certain TRBV genes (i.e., TRBV13 and TRBV19). However, CDR3β also plays an important role in MAIT TCR recognition since it is positioned directly above the antigen (47–49). We demonstrated CDR3β sequence hallmarks that can be used to distinguish MAIT from conventional T cells. We trained a classifier which has a high accuracy (ROC AUC ≈ 0.8) in this task and may be used for the annotation of MAIT sequences in bulk TCRβ sequencing data.

Contrary to MAIT cell subset proportions at steady state (17, 24, 50), the proportion of immature and MAIT1 cells was much higher in our dataset. This is probably related to the early time—7 wk after reconstitution—of the chimeras we studied, which would not have enough time to accumulate MAIT17 cells. This experimental setting recapitulates the early phase of MAIT cell development in young mice. It allowed us to capture some proliferating MAIT1 cells. Moreover, the even proportion of the different subsets (immature, MAIT1 and MAIT17 cells) increased the statistical power of our analysis on mature MAIT1 or MAIT17 cells. Still, it is very unlikely that MAIT subsets would interconvert in older mice and affect the conclusions of our analysis. Another dataset of scRNAseq and scTCRseq from Lee et al. (26) was obtained from older mice of 6 to 12 wk old and thus have a higher number of MAIT17 compared to MAIT1 clonotypes (577 vs. 93). Because of skewing to the MAIT17 subset, it is difficult to use this dataset to draw conclusions about TCR involvement and timing for MAIT fate choice.

One possible caveat regarding the clone fate tracking analysis is that TCRs with identical nucleotide sequences can theoretically be rearranged independently in different precursor cells. However, this is very unlikely as the absolute Pgen values of TCR nucleotide sequences found in both MAIT1 and MAIT17 subsets range from 2 × 10^−43^ to 3 × 10^−12^. Another mechanism that might lead to identical TCR sequences is an expansion of thymocytes at the CD4^−^CD8^−^ double negative stage during TCRβ selection. As Pgen for canonical MAIT TCRα sequences is ~8 × 10^−6^, the level of expansion required for this scenario is 10^5^-fold, which is biologically unrealistic. The number of MAIT1-MAIT17 clones is also high (n = 14) further supporting this result.

We generalized our conclusions to iNKT cells by analysis of scRNAseq and scTCRseq data from Lee et al. (26). We found that iNKT1 and iNKT17 TCR sequences form mixed clusters and cannot be distinguished based on their CDR3β. Moreover, we found two examples in which clones with the same amino acid but different nucleotide sequences committed to different fates. In silico simulation showed that there is a round of proliferation after iNKT fate choice. Although the Lee dataset was too sparse to confirm proliferation before commitment, taken together these observations indicate that our model of MAIT cell development is also relevant for iNKT cells.

Another approach to study the role of TCR characteristics in innate-like T cell commitment is retrogenic expression of the corresponding TCRs in mice. For iNKT cells Cruz Tleugabulova et al. (30) performed retrogenic expression of six iNKT TCRs displaying various affinities for the nominal ligand. No correlations between TCR avidity and iNKT subset composition were found to be statistically significant after correction for multiple comparison testing. Retrogenic TCR expression is low-throughput and may lead to artifacts due to expression of a rearranged TCR at an immature stage. In contrast, the approach presented in the current work, allows tracking hundreds of TCR clones in a natural polyclonal setting.

In our study, we used data from two mouse strains: B6 (which was used in our experiments) and BALB/c (used by Lee et al.). Although these two strains may differ in relative proportions of iNKT and MAIT subsets, these subsets share the same gene expression patterns as recently shown by Krovi et al. (41). All available scRNA-seq data for murine MAIT (23, 26, 51) and iNKT cells (25, 26, 52), obtained both from B6 (23, 25, 52) and BALB/c (26) mice were integrated. For both mouse strains, MAIT and iNKT subsets overlap in the same clusters. Thus, our results are relevant for both B6 and BALB/c mice.

Our conclusions about a fate choice being independent of TCR characteristics can be applied only to innate-like T cells—MAIT and NKT—in mice (in humans, effector MAIT cells have RORγt^+^T-bet^+^ phenotype). However, for other T cell subsets TCR characteristics might play a role in the fate commitment (53, 54), and the methodology developed in the current study could be applied to investigate this question. For this, scRNA-seq and scTCR-seq data should be obtained from a population of T cells which include both T cell subsets of interest and recognize one particular antigen (e.g., tetramer-sorted after vaccination; antigen with a limited diversity of specific TCR repertoire should be possibly chosen to increase chances to find pairs of TCRs with the same amino acid and different nucleotide sequence). Based on previous studies, TCR characteristics are likely to play a role in CD4+ vs. CD8+ lineage choice (4 to 7). CDR3 hydrophobicity promotes development of regulatory T cells (Treg) (1 to 3). These two cases illustrate two different ways TCR characteristics can be translated into distinct fates. For Treg vs. Tconv choice, selection is mediated by the same antigens, and higher affinity of TCR (which is correlated with hydrophobicity) leads to Treg fate. For CD4+ vs. CD8+ choice, the difference in TCR characteristics is due to restriction to MHC class II and class I molecules, respectively. T effector (Teff) vs. T follicular helper (Tfh) decision fate choice was shown to be correlated with TCR-peptide-MHC dwell time (55) and affinity (56). Regarding Th1 vs. Th2 lineage decision, it was initially hypothesized to be influenced by TCR avidity (57) but it is now thought that the choice is governed by cytokine environment and the type of interacting dendritic cells (58).

During their development in the thymus, immature MR1:5-OP-RU tetramer+ cells may be positively selected by either DP thymocytes or TECs, resulting in the acquisition of a MAIT (PLZF+) or “mainstream-like” (PLZF–) phenotype, respectively. Whether TCR characteristics are instructive or not in this choice cannot be addressed in the current study because we used bone marrow chimeras which lack mainstream-like MR1:5-OP-RU specific T cells.

Altogether, our results suggest the following four-stage model of MAIT cell development which is also relevant for iNKT cells:

1) MR1-expressing DP thymocytes mediate positive selection of immature MAIT cells;
2) An unknown endogenous (with also a possible role for exogenous 5-OP-RU) MR1-bound ligand induces the initial step of MAIT cell proliferation at the intermediate stage. This stage is also supported by a notable number of MAIT cells in germfree mice as compared to MR1-knockout mice (33).
3) Intermediate-stage cells commit to either MAIT1 or MAIT17 fate independently of TCR characteristics (possibly, being governed by cytokine environment). Another argument supporting TCR-independent fate commitment is the similar iNKT1:iNKT17 and MAIT1:MAIT17 cell proportions in germfree mice in which there is no preferential MAIT17 expansion;
4) Both MAIT1 and MAIT17 undergo an additional round of proliferation induced by gut microbiota-derived 5-OP-RU that reaches the thymus (33). Notably, the level of proliferation is higher for MAIT17 compared to MAIT1 cells which is supported by the higher MAIT17:MAIT1 cell proportion in mice from usual animal facilities and the higher expansion index in MAIT17 subset in our data.

## Methods

### Mice.

*Cd3e^−/−^Mr1^−/−^* mice were generated at Institut Curie by crossing, on a B6 background, MR1^−/−^ (16) with *Cd3e^−/−^* mice (59). B6-MAIT^Cast^ Rorc^GFP^ mice have been described elsewhere (11). All experiments were performed according to national guidelines and were approved by the relevant ethical committee (APAF1S no. 23976-2020020621584207 v2).

### Bone Marrow Chimera and Cell Preparation.

*Cd3e^−/−^Mr1^−/−^* mice were sublethally irradiated (420 rads) and reconstituted intravenously with 10^6^ bone marrow cells from B6-MAIT^Cast^ Rorc^GFP^ mice. Seven weeks after reconstitution, the thymus was harvested for further analysis of MR1:5-OP-RU restricted thymocytes. Single-cell suspension of thymus was obtained as described (17). MR1:5-OP-RU tetramer staining along with an anti-TCRb-APC (clone H57-597; 1:200) was performed at room temperature for 30 min in PBS 0.5% BSA 2 mM EDTA complemented with rat anti-mouse CD16/CD32 antibody and MR1:6FP to block nonspecific binding following the NIH Tetramer Core Facility instructions. Tetramer-based magnetic enrichment was performed as described (60). Cells were washed and stained for surface markers with following monoclonal antibodies: anti-CD44-APC-Cy7 (IM7, Biolegend; 1:400), -CD24-PE-Cy5 (M1/69, Biolegend; 1:1,600), -CD19-AF700 (ID3, Biolegend; 1:800), -B220-AF700 (RA3-6B2, eBioscience; 1:400), -CD11c-AF700 (N418, eBioscience; 1:200), -F4/80-AF700 (BM8, Biolegend; 1:200). For cell hashing in combination with 10× Chromium Single Cell 5′ Solution, TotalSeq-C anti-mouse Hashtags 2 to 7 (M1/42; 39-F11, Biolegend, 155863, 155865, 155867, 155869, 155871, 155873; 1:100 for all) were used according to manufacturer recommendations. Single-cell suspensions from the thymus of six hematopoietic chimera were pooled and DAPI (1:1000) staining was added just before FACS on an Aria III cell sorter (BD). Total TCRb+MR1:5-OP-RU+ cells were flow-sorted into PBS 0.35% BSA, centrifuged, counted, and 6,500 cells were loaded onto the Chromium 5′ chip. Reverse transcription, library preparation, and sequencing were performed according to manufacturer recommendations (10× Genomics).

### Single-Cell RNA-Seq Analysis.

Raw reads were processed using the Cell Ranger (version 6.0.0) The reference genome: mm10-2020-A for the scRNA-seq and vdj_GRCm38_alts_ensembl-3.1.0 for scTCR-seq. The median number of UMIs assigned to a TRA and TRB contig per cell was 5 and 24, respectively. All analyses were performed using R version 4.2.1 and the following packages: Seurat_4.1.3, clustree_0.4.4. Based on the distribution of the numbers of genes and molecules detected per cell, the following filters were applied to remove outliers: nFeature_RNA > 1,200 & nCount_RNA > 0. Cells containing more than 10% of mitochondrial genes were considered as dying cells and filtered out. For scRNA-seq analysis, all TCR-related genes were excluded in order to not interfere in the clusterization method (*Trav*, *Trbv*, *Trdc*, *Trdj*, *Trac*, *Traj*, *Trdd*, *Trgv*, *Trgc*, *Trgj*, *Trbd*, *Trbj*, *Trbc*). n = 2,000 highly variable features number was considered, graph-based clustering (Louvain method) was performed using the default parameters, and a UMAP (dims = 7) was constructed with a resolution of 0.5 based on the stability observed with the package clustree. The differentially expressed genes were determined using the FindAllMarkers() function (using a logistic regression base 10, Fold-change > 0.25).

Hash-tag oligos (HTO) were used to multiplex samples from six different mice. Dumultiplexing of HTO data was performed using CITE-seq-Count (version 1.4.3). HTO data were transformed using centered log-ratio (CLR) normalized and cell barcodes were assigned to mice of origin using Seurat function HTODemux() with default threshold for classification (positive.quantile = 0.99). Cells annotated as “negative” and “doublets” based on HTO array were excluded from the analysis. In total, 3,113 cells with a barcode detected in both RNA and HTO arrays and annotated as HTO singlets were considered for downstream analysis.

Integration of scRNA-seq datasets from our study and Lee et al. (26) was performed using Harmony (61), which was shown to be the best method for such task in a recent benchmark (62).

### iNKT Dataset.

The raw fastq files for TCRαβ-seq single-cell dataset of thymic iNKT cells from BALB/c mice from Lee et al. (26) (PRJNA549112) were downloaded. The data were composed of two biological replicates (1,848 and 2,325 cells) with three technical replicates each containing a one-to-one mixture of NKT, γδT, and MAIT cells. The reads were aligned and feature-barcode matrices were generated as above. Two biological replicates were analyzed independently, for each aggregated expression from the three technical replicates was considered (AggregateExpression() from seurat package). For scRNA-seq analysis, TCR genes were excluded from RNA-seq analysis as above. TCR information was added to the Seurat meta-data object; iNKT cells were identified based on TRAV11 expression. Based on the distribution of the numbers of genes and molecules detected per cell, the following filters were applied to remove outliers: nCount_RNA ≥ 7,500 & nFeature_RNA ≥ 1,000 & nCount_RNA ≤ 90,000. Cells containing more than 10% of mitochondrial genes were filtered out. The number of highly variable features considered for replicates 1 and 2 was 2,500 and 3,000, respectively. Graph-based clustering (Louvain method) was performed using the default parameters, and a UMAP (dims = 15 and dims = 20) was constructed with a resolution of 0.5 based on the stability observed with the package clustree. Cluster assignment to iNKT populations was done based on expression of marker genes (NKT1: *Tbx21*, *Nkg7*; NKT17: *Rorc*; intermediary: *Zbtb16*; immature: *Dntt*, *Rag1*, *Itm2a*).

### TCR Repertoire Analysis.

The fractions of cells with different numbers of detected TCRα and TCRβ chains (0, 1, or 2) are shown in *SI Appendix*, Fig. S15. For the majority of cells (56%) 1 TCRα and 1 TCRβ were detected. A number of cells contained either 0 TCRα and 1 TCRβ (22%) or 1 TCRα and 2 TCRβ (14%) or 2 TCRα and 1 TCRβ (2%). Cells with two detected TCRβ chains were excluded from the analysis since they probably correspond to doublets. Cells with 0 TCRα were excluded to avoid biases coming from the potential pairing of the same TCRβ with different TCRα in different cells, which may stem from either convergent TCRβ recombination or TCRβ expansion during beta selection at the CD4^-^CD8^-^ stage. Among 71 cells expressing 2 TCRα and 1 TCRβ, we excluded those for which both TCRα chains are either TRAV1^−^ (n = 1) or TRAV1^+^ (n = 42; probably representing doublets with a dropout of one of the two TCRβ chains). For the remaining 28 dual TCRα-expressing cells, only TRAV1^+^ TCRα chain was considered as defining MR1-tetramer specificity. For TCR repertoire analysis, we considered 1,797 cells with strictly 1 TCRα and 1 TCRβ and 28 cells with 2 TCRα and 1 TCRβ chains.

For TCR Repertoire Analysis We Used the Following Definitions.

A “clonotype” is a set of cells which have identical amino acid sequence of both TCRα and TCRβ chains. Within a clonotype, the nucleotide sequence and the mouse of origin may be different for different cells.

A “clone” is a set of cells which have identical nucleotide sequence of both TCRα and TCRβ chains and originated from the same mouse. Given the low generation probability of every particular nucleotide sequence, we assume that all cells within the clone originated from a single precursor.

Clonotypes and clones were assigned to groups: MAIT1 if they included at least one MAIT1 cell and no MAIT17 cells; MAIT17 if they included at least one MAIT17 cell and no MAIT1 cells; MAIT1-MAIT17 if they included at least one MAIT1 cell and at least one MAIT17 cell; other if they included only immature, intermediate and cycling MAIT cells and no mature MAIT1 or MAIT17 cells.

### Classifier to Distinguish between MAIT Subsets and Control Clonotypes.

TCRβ sequences of control clonotypes were taken either from the publication of Sethna et al. (35) (results are shown in Fig. 4 *B* and *C*), or from the dataset published by the Chudakov laboratory [https://zenodo.org/record/6339774#.ZAikGXbMJPa] (results are shown in *SI Appendix*, Fig. S5 *B* and *C*).

To build a classifier, we considered only CDR3β sequences, since the use of the α chain information (which is invariant for MAIT cells) would make the task too trivial.

To make the comparison between the performance of different classifiers fair, for the construction of a training dataset for all three groups (MAIT1, MAIT17 and control CDR3β sequences) we considered the same number of clonotypes (n = 300) randomly chosen from the full dataset. The validation of the classifier was performed in two alternative ways:

1) Fivefold cross-validation: Training data were split into five equal parts, and then, the classifier was trained on four of them and tested on the remaining one which was not used for training. The procedure was repeated for each of the five parts and then the performance metrics were averaged;
2) The classifier was trained using our own dataset as training and then performance was assessed using the independent dataset from Lee et al. (26). Test control sequences were randomly chosen from Sethna dataset (or Chudakov’s one when specifically stated) without intersection with sequences from the training dataset.

For MAIT cells, both validation approaches were tested. For iNKT cells, only fivefold cross-validation was used since only a single scTCR-seq dataset for iNKT cells from Lee et al. (26) is available.

The classifier was constructed based on SONIA software (36), which uses one-hot encoding of the sequence. That means that each CDR3β sequence was encoded by a set of features, each of which corresponds to a particular amino acid in a particular position and has a value of 1 if the given amino acid is observed in the given position and a value of 0 otherwise. To account for sequences of different lengths, SONIA encodes sequences in both forward and reverse directions (in the latter case, the position is defined as “−1,” “−2,” etc.). After sequence encoding, a logistic regression model was trained on the training data; L1 (weight 10^−4^) and L2 (weight 10^−4^) regularization was applied to avoid overfitting as in ref. 6.

### Simulations.

#### *Role of TCR characteristics in fate choice*.

If fate choice is influenced by TCR characteristics, TCRs with identical amino acid sequence independently recombined in distinct progenitor cells would preferentially commit to the same fate (either MAIT1 or MAIT17). To quantify this, we performed in silico simulations of a scenario in which fate choice is not dependent on TCR characteristics. For this scenario, we performed two simulations. First, we focused on clonotypes which consist of several clones (Fig. 2*A*; only MAIT1 and MAIT17 clones were considered). *N_obs_* = 15 out of total 26 such clonotypes included both MAIT1 and MAIT17 clones (referred to as “mixed clonotypes”). Then we performed MAIT subset identity label shuffling as shown in *SI Appendix*, Fig. S16 simulating commitment of each clone to either MAIT1 or MAIT17 subset independently of other clones (MAIT subset ratio and distribution of the number of clones within clonotypes were preserved). This reshuffling procedure was repeated 1,000 times and after each iteration, we calculated the number of mixed clonotypes *N_exp_* based on the resulting shuffled dataframe. The distribution of *N_exp_* had median 14 with 95% CI from 9 to 19. *N_obs_* fits well to this interval, indicating that TCR characteristics are not instructive for MAIT fate choice. The second simulation was performed for clonotypes detected both in our and Lee datasets. Only MAIT1 and MAIT17 clonotypes were considered. *N_obs_* = 13 out of total 19 such clonotypes were assigned to MAIT1 in one dataset while MAIT17 in the other. We then performed label identity shuffling analogously as described above. The procedure was repeated 1,000 times and after each iteration, we calculated the number of clonotypes assigned to different subsets in the two datasets *N_exp_*. The obtained *N_exp_* distribution ranged from 3 to 16. *N_obs_* fits well to this interval, indicating that TCR characteristics are not instructive for MAIT fate choice.

#### *Timing of proliferation in relation to fate commitment*.

To explore whether there is a proliferation stage before fate commitment, we first considered a scenario in which no proliferation occurs before fate choice. In this scenario, every single precursor cell first commits to some fate immediately after positive selection (e.g., at the moment of TCR interaction with MR1-ligand complex on DP thymocytes) and starts to proliferate only after commitment; thus we expect that all descendants of a single precursor would commit to the same fate. Indeed, in this scenario, no clones including both MAIT1 and MAIT17 cells can exist. However, in our experimental dataset, we detected n = 14 cases when a single precursor cell gave rise both to MAIT1 and MAIT17 descendants (in other words, clones including both MAIT1 and MAIT17 cells). This result indicates that the underlying assumption (no proliferation before fate choice) is false. Based on this reasoning, we included a first round of proliferation (before fate commitment) to our model.

To explore whether there is a proliferation stage after fate commitment, we performed an in silico simulation of a scenario in which no proliferation occurs after fate choice: a precursor cell is first positively selected by MR1, then undergoes proliferation, and finally chooses its fate with subsequent maturation occurring without additional cell proliferation. In this case, the subset identity of each cell is independent of other cells. We simulated this scenario by random assignment of subset identity to each cell in our dataset while preserving the total number of cells, the relative ratio between MAIT subsets, and the distribution of clonotype sizes. The simulation was performed in the following way. First, based on the analysis of experimental scRNA-seq and scTCR-seq data, we assembled a dataframe with three columns: 1) cell ID, 2) merged amino acid TCRα+TCRβ sequence (using of nucleotide sequence here does not change the conclusions), 3) MAIT subset identity. Then we randomly shuffled the third column. This procedure does not affect the number of cells (as all the rows are kept), the clonotype size distribution (as the TCR sequence column is not modified), and the subset distribution (as the subset ID column is only shuffled without changing the values). This reshuffling procedure was repeated 1,000 times and after each iteration, we calculated the number of MAIT1-MAIT17 clonotypes based on the resulting shuffled dataframe. In this way, we obtained the distribution of expected number of MAIT1-MAIT17 clonotypes (*N_exp_*; median: 56; 95% CI: 45 to 68) under the assumption of no proliferation occurring after fate commitment. Then, we used the original dataframe without reshuffling of MAIT subset identity column to calculate the experimentally observed number of MAIT1-MAIT17 clonotypes (*N_obs_* = 25). This number is significantly lower than in the *N_exp_* distribution, allowing us to reject the possibility that there is no proliferation after the fate commitment. Based on this simulation, we include the second round of proliferation (after fate commitment) to our model.

## Supplementary Material

Appendix 01 (PDF)

## Data Availability

Sequencing data have been deposited in NCBI's Gene Expression Omnibus and are accessible through https://www.ncbi.nlm.nih.gov/geo/query/acc.cgi?acc=GSE236666 (GSE236666). All the code and data required to reproduce the analysis performed in the study are available at GitHub repository: https://github.com/vadim-karnaukhov/MAIT_TCR. Previously published data were used for this work (26).
