# Supplementary material for: Innate-like T cell subset commitment in the murine thymus is independent of TCR characteristics and occurs during proliferation
Source: Proc Natl Acad Sci U S A. 2024 Mar 26;121(14):e2311348121. doi: 10.1073/pnas.2311348121 (PMC10998581; doi:10.1073/pnas.2311348121)
Supplement: Supplementary file 1 — Appendix 01 (PDF) [file pnas.2311348121.sapp.pdf]

Supplementary Figures

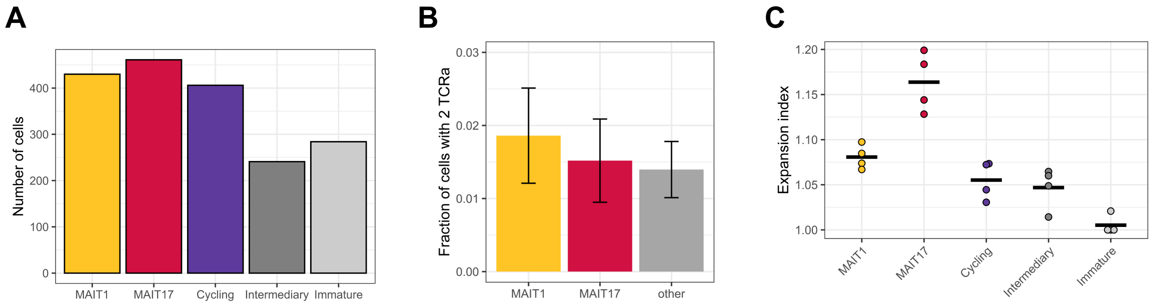

Supplementary Figure 1. A. Number of cells corresponding to each of the MAIT subpopulations in the dataset. **B.** Fraction of cells expressing two TCRα chains in different subsets. Whiskers show standard deviation. **C.** Expansion levels for different MAIT subsets. The expansion index was calculated as the mean clone size within the given subset (i.e. the mean number of cells having identical TCRα+TCRβ nucleotide sequence within the given subset for a given mouse). Each dot corresponds to one mouse. Downsampling to the equal number of cells in each subset in each mouse (n = 43) was performed.

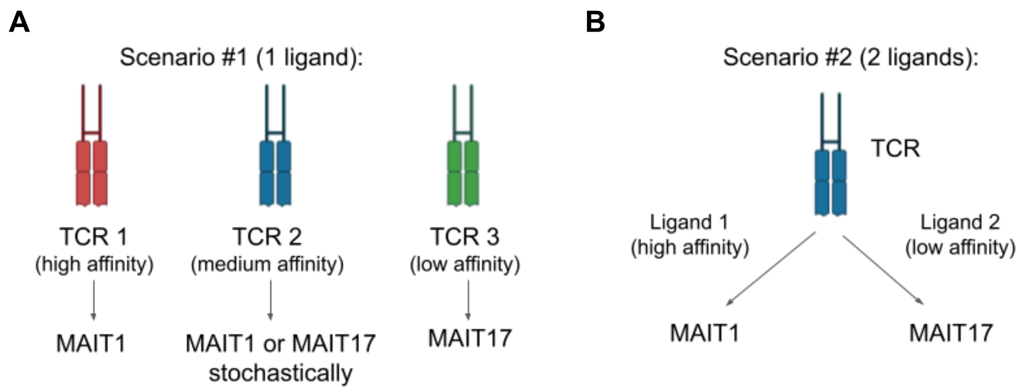

Supplementary Figure 2. Scenarios with TCR affinity-instructed lineage choice resulting in MAIT1-MAIT17 clonotypes. A. Scenario with a single MAIT ligand. **B.** Scenario with two MAIT ligands.

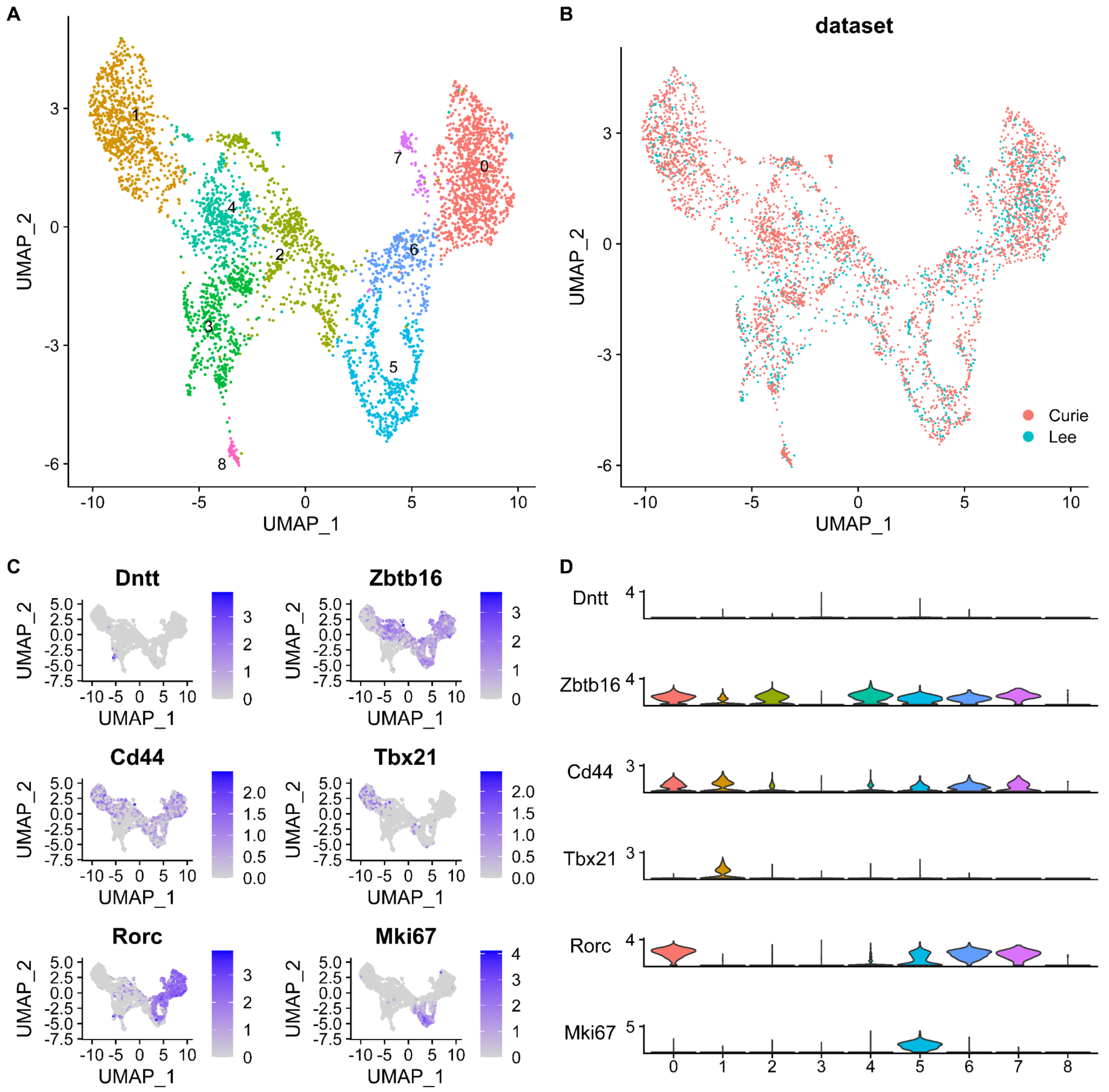

Supplementary Figure 3. Integration of scRNA-seq datasets of MAIT cells from Lee et al. and our study. A. UMAP of MAIT cell populations. **B.** UMAP stlited according to the dataset of origin. **C-D.** Expression of marker genes projected on the UMAP (C) and shown as a violin plot (D).

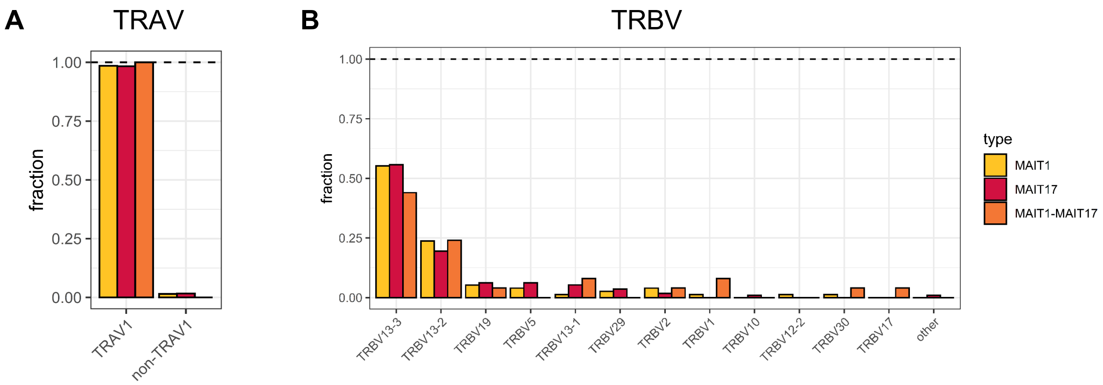

Supplementary Figure 4. TRAV (**A**) and TRBV (**B**) gene usage for MAIT1 (yellow), MAIT17 (red) and MAIT1-MAIT17 (orange) clonotypes.

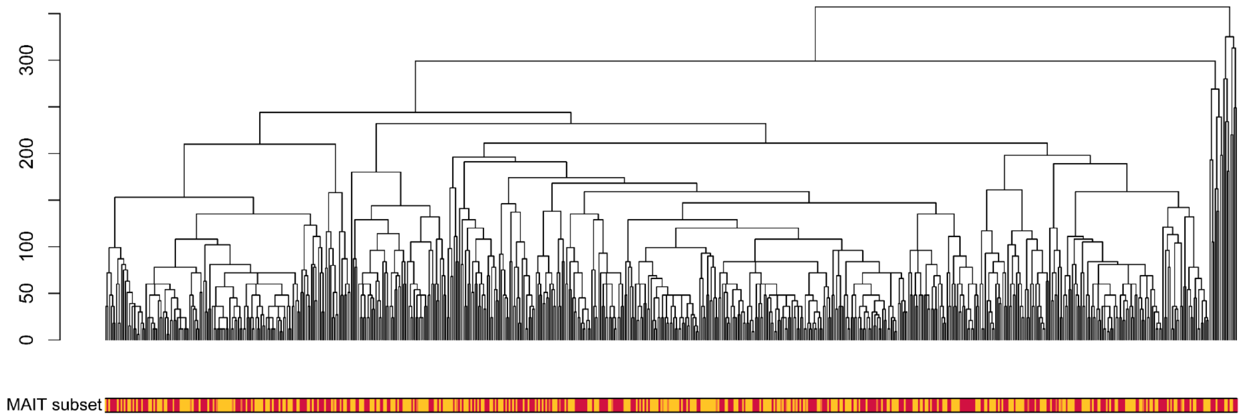

Supplementary Figure 5. Dendrogram for TCR sequences of MAIT1 and MAIT17 clonotypes. Y axis corresponds to the distance between TCR sequences that was calculated using tcrdist3 package (35). The full TCR sequences were considered (both TCRα and TCRβ chains, including CDR1, CDR2 and CDR3). Clustering was performed using stats::hclust() function in R. Each leaf of the dendrogram corresponds to a single clonotype sequence whose subset identity is color-coded in the panel below the dendrogram (yellow for MAIT1 and red for MAIT17).

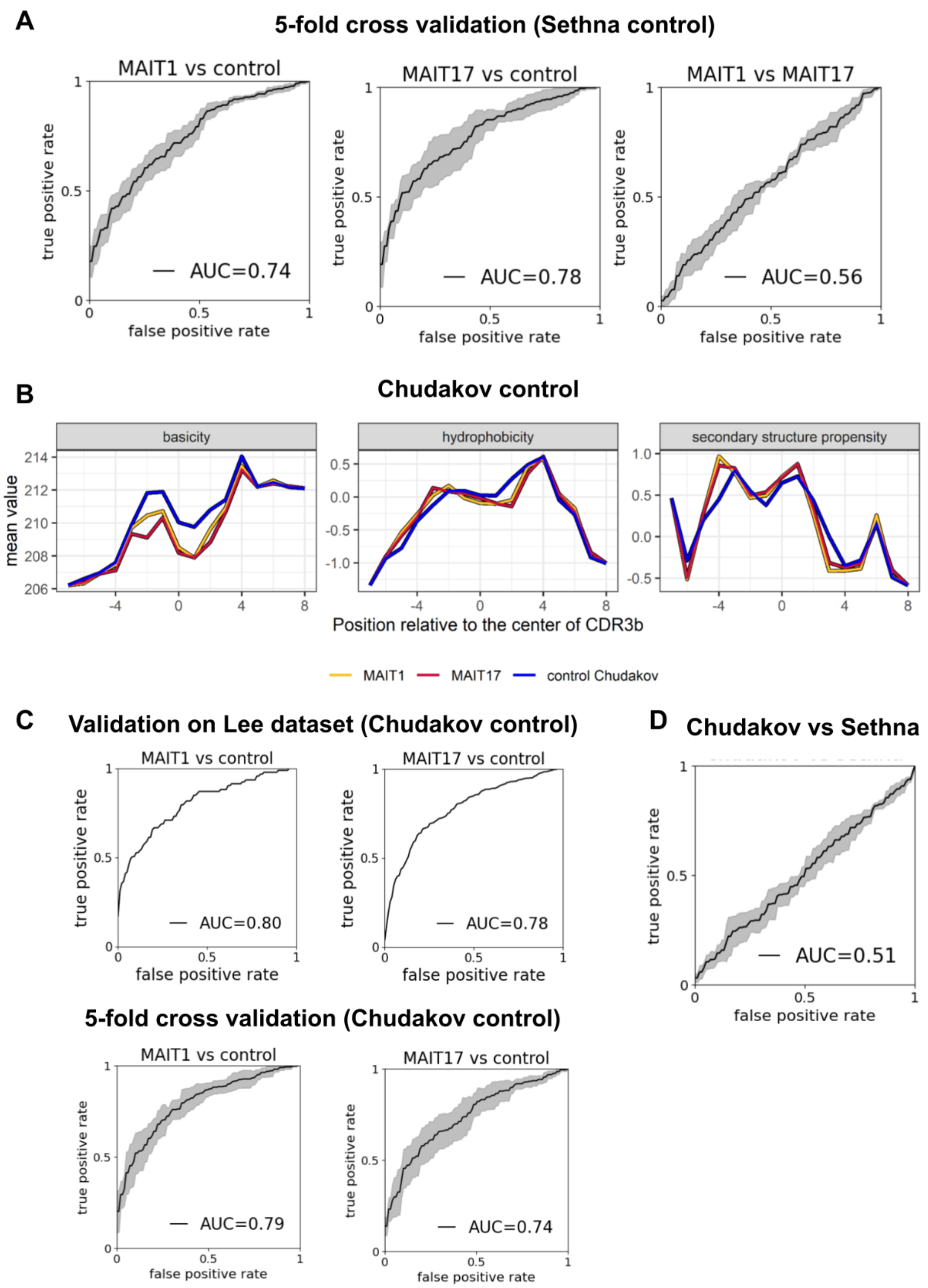

Supplementary Figure 6. Comparison of MAIT1 and MAIT17 with control sequences from different sources. A. 5-fold cross validation of SONIA classifier from Fig. 4E. **B.** Physicochemical profiles of CDR3β sequences of MAIT1 and MAIT17 compared to control sequences from Chudakov dataset (analogous to Fig. 4B). **C.** ROC curves analogous to the ones in Fig. 4C and Sup. Fig. 6A with the use of control sequences from Chudakov. **D.** ROC curve for distinction between control sequences from Sethna and Chudakov.

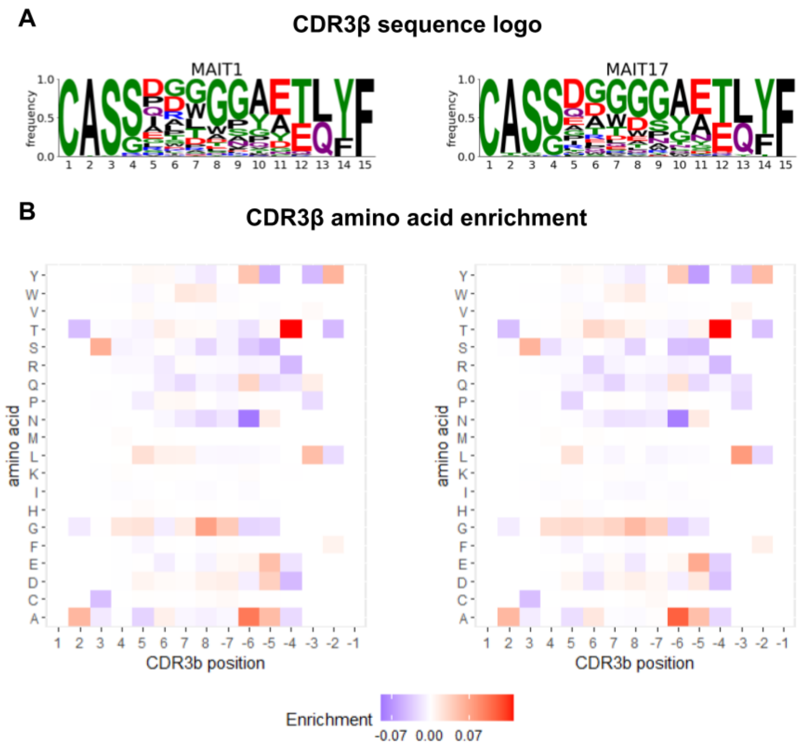

Supplementary Figure 7. CDR3β sequence and enrichment logo for MAIT1 and MAIT17. A. Sequence logo is plotted as fractions of different amino acids in particular CDR3β positions. **B.** Relative enrichment of positional amino acid usage is calculated as $w_{MAIT} - w_{control}$, where w_{MAIT} and $w_{control}$ are weights associated to amino acid at the given position by SONIA model for MAIT or control synthetic repertoires, respectively.

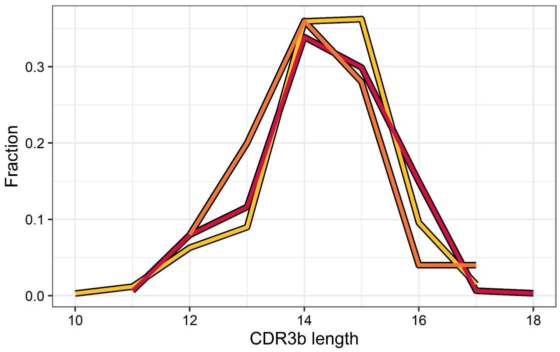

Supplementary Figure 8. Distribution of CDR3β lengths for MAIT1 (yellow), MAIT17 (red) and MAIT1-MAIT17 (orange) clonotypes.

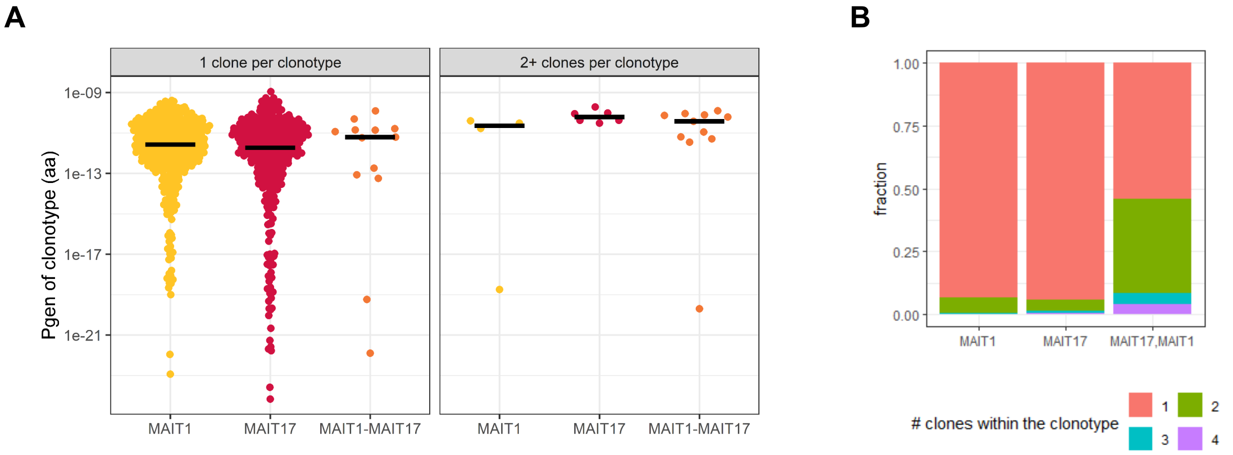

Supplementary Figure 9. MAIT1-MAIT17 clonotypes are preferentially composed of sequences independently rearranged in distinct precursors. A. Pgen of clonotypes, grouped by the number of clones comprising them. Black line = Median. **B.** Each clonotype is annotated based on the number of clones it includes. Then the percentage of clonotypes composed of different numbers of clones is calculated for MAIT1, MAIT17 and MAIT1-MAIT17 clonotypes.

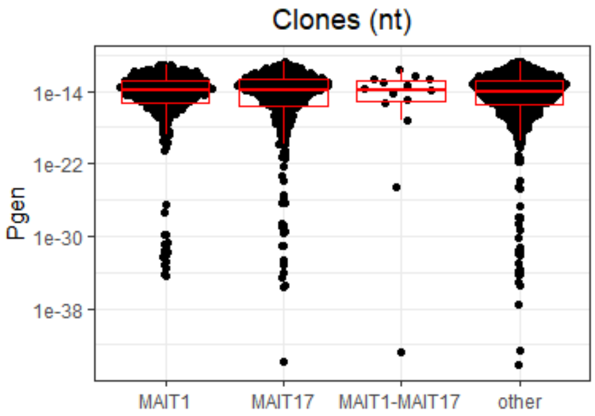

Supplementary Figure 10. Probability of generation of nucleotide CDR3α+CDR3β sequence of MAIT clones.

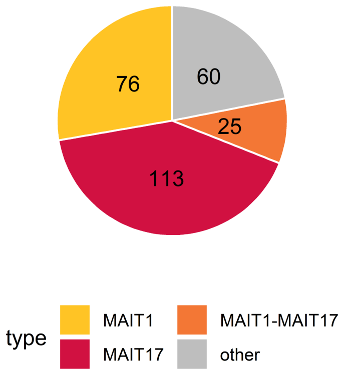

Supplementary Figure 11. Conotype distribution across MAIT subsets. Number of “MAIT1”, “MAIT17” and “MAIT1-MAIT17” clonotypes as well as clonotypes containing neither MAIT1 nor MAIT17 cells (“other”). Clonotypes with ≥2 cells (n = 273) are shown.

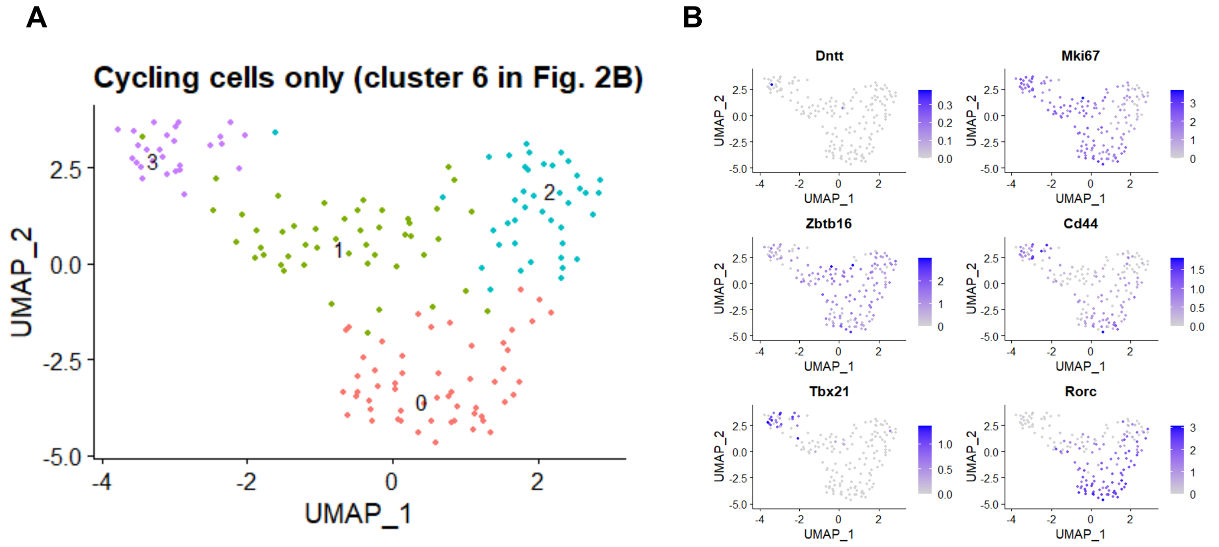

Supplementary Figure 12. MAIT cells proliferate at different stages of development. Re-clustering was performed for cells from cluster #6 (Fig. 1B), which had the highest expression level of the proliferation marker *Mki67*. **A.** UMAP. **B.** Expression of marker genes. Cells in cluster #0 correspond to MAIT17 cells, in clusters #1 and #2 – to intermediate-stage MAIT cells, in cluster #3 – to MAIT1 cells.

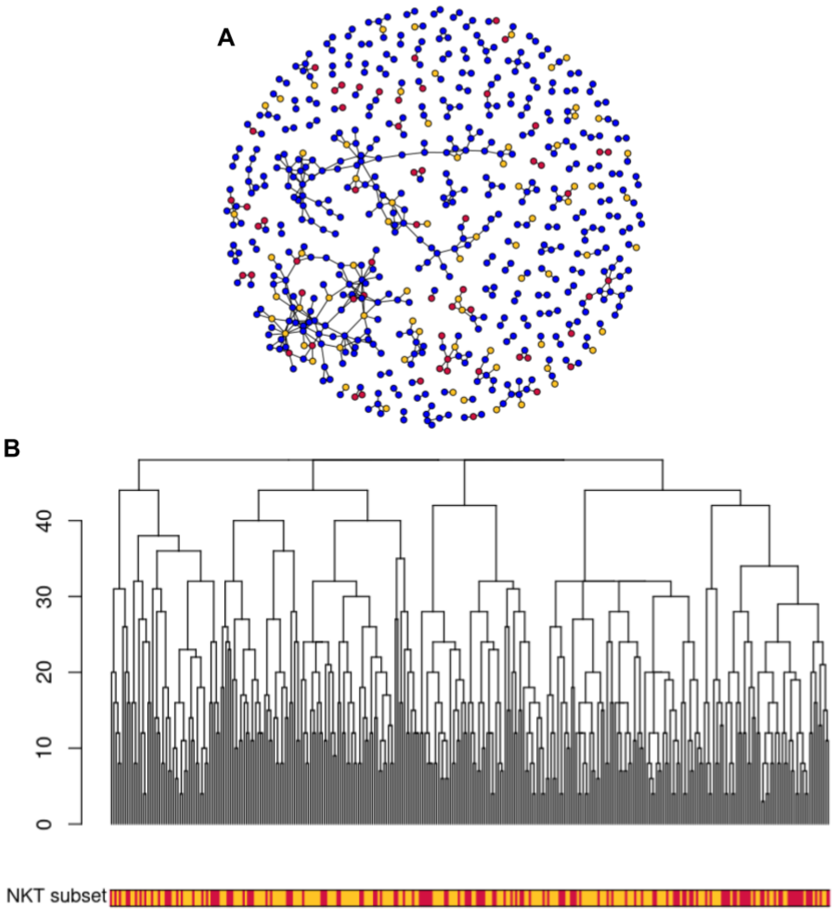

Supplementary Figure 13. Sequence similarity of NKT1 and NKT17 clonotypes. A. Graph analogous to Fig. 4A and Fig. 4D, showing sequence similarity of NKT1 (yellow), NKT2 (blue) and NKT17 (red) clonotypes. **B.** Denderogram of CDR3β sequences of NKT1 and NKT17 clonotypes, analogous to Sup. Fig. 4. Distances are calculated using tcrdist3.

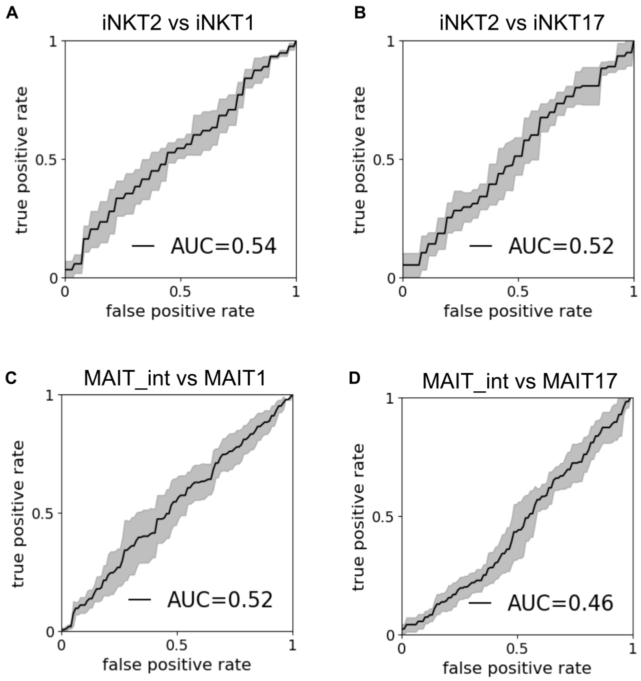

Supplementary Figure 14. Performance of SONIA-based classifier to distinguish TCR CDR3β sequences from iNKT2 vs iNKT1 (A), iNKT2 vs iNKT17 (B), intermediate-stage MAIT (MAIT_int) vs MAIT1 (C), MAIT_int vs MAIT17 (D). ROC AUC value for 5-fold cross-validations are shown. For MAIT cells our dataset was used (as Lee et al. dataset contains too few MAIT1 cells), for iNKT cells Lee et al. dataset was used.

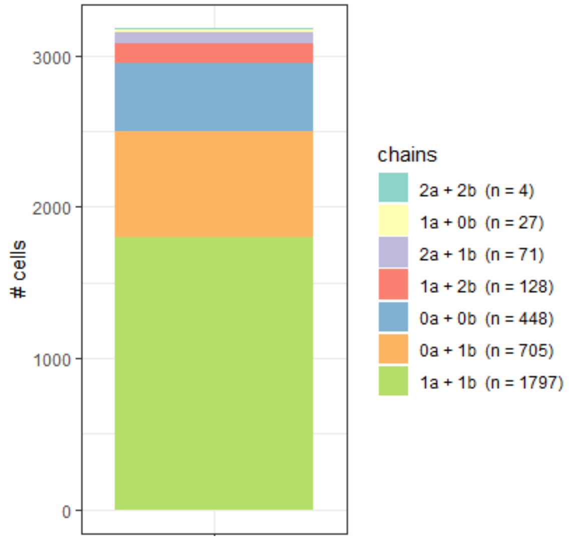

Supplementary Figure 15. Number of cells with different numbers of detected TCRα and TCRβ chains in our dataset.

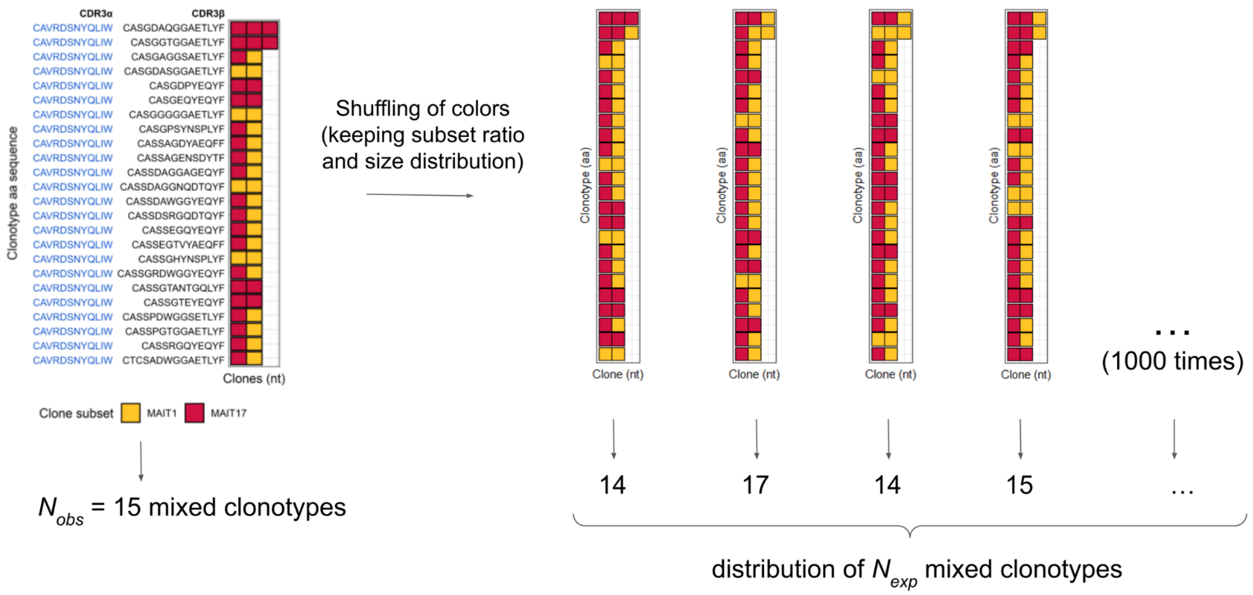

Supplementary Figure 16. Illustration of a label shuffling simulation for clonotypes made of several clones.
